# Early-life serological profiles and the development of natural protective humoral immunity to *Streptococcus pyogenes* in a high-burden setting

**DOI:** 10.1038/s41591-025-03868-4

**Published:** 2025-08-08

**Authors:** Alexander J. Keeley, Fatoumata E. Camara, Edwin P. Armitage, Gabrielle de Crombrugghe, Jainaba Sillah, Modou Lamin Fofana, Victoria Rollinson, Elina Senghore, Musukoi Jammeh, Alana L. Whitcombe, Amat Bittaye, Haddy Ceesay, Isatou Ceesay, Bunja Samateh, Muhammed Manneh, Martina Carducci, Luca Rovetini, Elena Boero, Luisa Massai, Lady Chilel Sanyang, Ousman Camara, Ebrima E. Cessay, Miren Iturriza, Danilo Gomes Moriel, Adam Kucharski, Pierre R. Smeesters, Anne Botteaux, Ya Jankey Jagne, Nicole J. Moreland, Ed Clarke, Beate Kampmann, Michael Marks, Omar Rossi, Henrik Salje, Claire E. Turner, Thushan I. de Silva

**Affiliations:** 1 Vaccines and Immunity Theme, MRC Unit The Gambia at the London School of Hygiene and Tropical Medicine, Fajara, The Gambia; 2 Division of Clinical Medicine, School of Medicine and Population Health, University of Sheffield, Sheffield, UK; 3 The Florey Institute of Infection, University of Sheffield, Sheffield, UK; 4 Department of Clinical Research, London School of Hygiene and Tropical Medicine, London, UK; 5 Population Health Sciences, Bristol Medical School, University of Bristol, Bristol, UK; 6 Molecular Bacteriology Laboratory, European Plotkin Institute for Vaccinology, Université Libre de Bruxelles, Brussels, Belgium; 7 Department of Paediatrics, Brussels University Hospital, Academic Children Hospital Queen Fabiola, Université Libre de Bruxelles, Brussels, Belgium; 8 Faculty of Medical and Health Sciences, The University of Auckland, Auckland, New Zealand; 9 GSK Vaccines Institute for Global Health (GVGH), Siena, Italy; 10 Centre for Mathematical Modelling of Infectious Diseases, London School of Hygiene and Tropical Medicine, London, UK; 11 Charité Centre for Global Health, Charité – Universitätsmedizin Berlin, Berlin, Germany; 12 Hospital for Tropical Diseases, University College London Hospital, London, UK; 13 Division of Infection and Immunity, University College London, London, UK; 14 Department of Genetics, University of Cambridge, Cambridge, UK; 15 School of Biosciences, University of Sheffield, Sheffield, UK

**Keywords:** Bacterial infection, Bacteriology, Translational immunology

## Abstract

*Streptococcus pyogenes* leads to 500,000 deaths annually, many due to rheumatic heart disease in low-income settings. Limited understanding of natural protective immunity to *S. pyogenes* hinders vaccine development. Here we describe the evolution of serological profiles to conserved vaccine antigens and serotype-specific M proteins from birth and throughout the life course in The Gambia. As placentally transferred IgG waned after birth, serological evidence of new exposure was seen in 23% of infants during the first year of life. After culture-confirmed *S. pyogenes* events, the highest IgG increases occurred in children younger than 2 years of age after both pharyngeal and skin disease and asymptomatic carriage at both sites. Higher IgG levels against conserved vaccine antigens correlated with functional activity and were associated with protection from culture-confirmed events after adjustment for age and anti-M protein IgG levels. To our knowledge, our data provide the first evidence of protection associated with humoral immunity to conserved vaccine candidate antigens in humans.

## Abstract (web summary)

Serological profiles from two observational cohort studies in The Gambia characterize surrogates of protection against *Streptococcus pyogenes* and reveal an antibody response against antigens that are currently in vaccine development.

## Main

*S. pyogenes* (Group A *Streptococcus*) is a major global pathogen responsible for 500,000 deaths annually, with the majority due to rheumatic heart disease (RHD) caused by long-term pathological immune sequelae^1,2^. An effective and equitable *S. pyogenes* vaccine is a global priority^3^, yet few candidates are currently in clinical development^3,4^. In addition to preventing invasive infections, an *S. pyogenes* vaccine needs to prevent throat and skin (pyoderma) infections, and perhaps asymptomatic carriage in children, that lead to pathological immune priming responsible for RHD^5^. Much of the *S. pyogenes* disease burden, including RHD, is experienced in low- and middle-income countries (LMICs), where skin infections are more common than in high-income countries (HICs)^1,2,6^.

A recognized scientific barrier to developing an *S. pyogenes* vaccine is the lack of understanding of naturally occurring immunity, particularly to protect against pharyngitis and pyoderma, which represent endpoints for future vaccine trials^7,8^. The prevalence of *S. pyogenes* pharyngitis and pyoderma progressively reduces from childhood to adulthood, suggesting that naturally protective immunity is acquired through repeated exposures^9,10^. Moreover, pooled intravenous immunoglobulin (IVIG) can promote opsonization, phagocytosis and killing of bacteria in vitro^11,12^. Together, these findings suggest that naturally occurring humoral immunity to *S. pyogenes* is one mechanism protecting adults from infection.

*S. pyogenes* vaccine development has taken two broad approaches. Initial efforts focused on the M protein, a major surface-expressed virulence factor encoded by the *emm* gene, also used for strain typing^3,13,14^. The M protein is immunogenic, with type-specific antibodies shown to protect in animal models and in limited observational human studies^15–19^. With over 275 *emm* types, multivalent M protein vaccines are limited by the high *emm*/M type diversity in LMICs^20,21^. An alternative approach focuses on conserved antigens such as the *S. pyogenes* cell envelope protease (SpyCEP), *S. pyogenes* adhesion and division protein (SpyAD), Streptolysin O (SLO) and the streptococcal Group A carbohydrate (GAC)^22^. Although animal models and genomic analyses demonstrate a promising role for these as vaccine antigens, their contribution to natural protection in humans remains unknown^22–27^. With both multivalent M protein and conserved antigen vaccines in development, understanding the evolution of natural immunity to these different antigens in early life and their relative roles in protection remains vital.

Within prospective observational cohort studies in The Gambia spanning the entire life course, we characterize serological profiles to leading conserved *S. pyogenes* vaccine antigens and *emm* type-specific M protein hypervariable regions. We describe the age-related, carriage-related and disease-related changes in antibody levels. We demonstrate, to our knowledge for the first time, correlates of protection against natural *S. pyogenes* infection associated with antibodies to conserved vaccine antigens.

## Results

### Study cohorts and *S. pyogenes* events

**Fig. 1:**
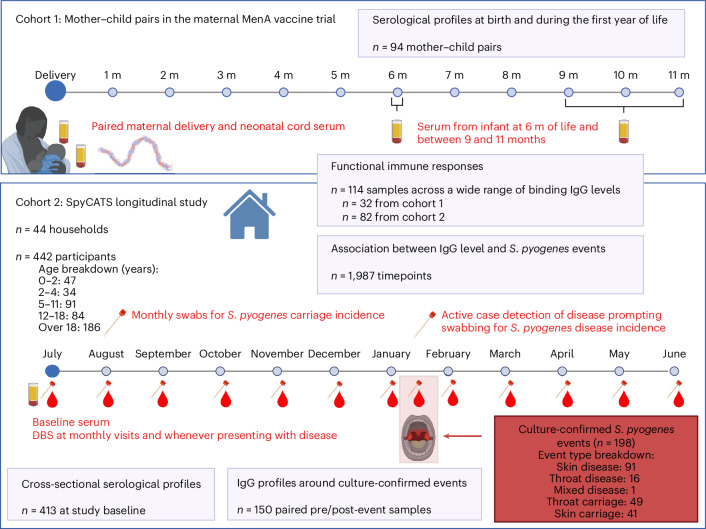
Study design and participants. Cohort 1 consisted of 94 mother–child pairs from The Gambia recruited to a maternal vaccination trial with meningococcal conjugate vaccine. The newborn infants were followed through the first year of life. No microbiological sampling was performed on cohort 1. Cohort 2 comprised participants in the SpyCATS household cohort study. Red text indicates the sampling framework within both cohorts. In cohort 2, participants were swabbed from normal throat and skin to detect carriage. Participants could report disease symptoms (sore throat and skin sores) to the study team, prompting swabbing from the relevant site to detect disease events. Antibodies were measured from serum collected at study baseline and from DBSs at monthly visits and at any disease presentation. Purple boxes represent the number of samples included in each analysis. Breakdown of age groups and event types from cohort 2 is provided. Created in BioRender.com. m, months.

The dynamics of *S. pyogenes*-specific antibodies, carriage and disease events were measured in a prospective, household cohort study conducted in the urban area of Sukuta, The Gambia, over a 13-month period in 2021–2022 (*S. pyogenes* carriage acquisition, persistence and transmission dynamics within households in The Gambia (SpyCATS), NCT05117528)^10^. In total, 442 individuals from 44 households were recruited and followed-up at monthly visits, comprising 256 children younger than 18 years of age (58%) and a median age of 15 years (range, 0–85; interquartile range (IQR), 6–10), 233 (58%) female participants and a median household size of seven (IQR, 6–10). Participants were also seen between monthly visits if new symptoms consistent with pyoderma or pharyngitis were reported. Incidence and prevalence of *S. pyogenes* in this cohort were reported previously^10^. In total, 108 *S. pyogenes* disease events (16 pharyngitis, 91 pyoderma, 1 mixed) and 90 carriage events (49 pharyngeal, 41 skin) were identified by bacterial culture in 141 individuals during the study (Supplementary Fig. 1). For greater resolution of antibody dynamics during the first year of life, serum samples from 94 mother–child pairs recruited to a clinical trial of meningococcal conjugate vaccine in pregnancy in 2018–2019 at an urban clinic in The Gambia (NCT03746665) were additionally included. The median age of mothers was 26 years (IQR, 23–29), with 35 (37%) female children. Figure 1 provides an overview of the study design.

### Early-life serological profiles

**Fig. 2:**
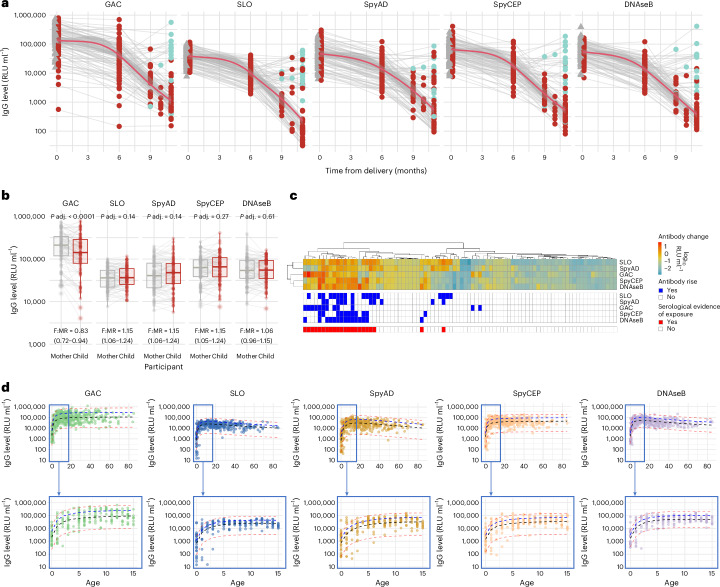
Early-life serological profiles and maternal antibody transfer. **a**, Longitudinal IgG antibody profiles from mother–infant pairs (*n* = 94) over the first year of life. Gray triangles represent maternal delivery samples; dots represent infant samples. Red dots denote no observed titer increase between 6 months and subsequent visits; green dots indicate serological evidence of exposure (IgG rise to ≥2 antigens or >0.5 log_10_ RLU ml^−1^ to one antigen) between 6 months and subsequent visit. The red line shows the mean with 95% CI (LOESS method). **b**, Paired maternal and cord blood IgG levels at delivery (*n* = 94). Box plots show medians, IQR and 1.5× IQR whiskers; outliers are plotted individually. Two-sided paired Wilcoxon signed-rank tests were used, and *P* values were adjusted using FDR correction. F:MR IgG transfer ratios are shown. **c**, Antibody dynamics between 6 months and 9–11 months in infants with complete data (*n* = 86). Each column represents an individual participant. Top panel shows absolute change in log_10_ IgG levels between 6 months and 9–11 months. Hierarchical clustering using Euclidean distance and complete linkage was performed across individuals (columns) and antigens (rows). Middle panel shows binary changes (blue, increase; white, decrease). Bottom panel highlights infants with serological evidence of exposure. **d**, Cross-sectional IgG levels by age (*n* = 413, 0–85 years). Median (black), 80th (blue) and 2.5th/97th centile (red) lines were modeled using fractional polynomials. The bottom panel focuses on children aged 0–15 years. LOESS, locally weighted scatterplot smoothing; *P* adj., adjusted *P* value.

IgG specific to the conserved vaccine antigens GAC, SLO, SpyAD and SpyCEP was measured from mother–child pairs in paired neonatal cord and maternal serum samples at delivery and in serum during the first year of life, along with DNAseB antibodies that are additionally used as serological evidence of *S. pyogenes* infection^28,29^. Antigen-specific antibody levels were quantified against an IVIG standard curve and expressed as relative Luminex units per milliliter (RLU ml^−1^). Efficient placental transfer of *S. pyogenes*-specific IgG was observed, with no difference between paired maternal and cord sera at delivery in levels of IgG against SLO, SpyAD, SpyCEP and DNAseB (*P* > 0.1 for all; Fig. 2a,b). GAC-specific IgG was lower in cord sera compared to maternal samples (5.08 versus 5.25 log_10_ RLU ml^−1^, *P* < 0.0001; Fig. 2a,b), with lower fetal:maternal transfer ratios (F:MRs). Waning of *S. pyogenes* antigen-specific IgG was observed in most children during the first 11 months of life (Fig. 2a). Between 6 months and the subsequent sample (9, 10 or 11 months), 22 infants (23%) demonstrated serological evidence of new *S. pyogenes* exposure, defined as any increase in IgG level to two or more antigens or greater than 0.5 log_10_ RLU ml^−1^ increase to a single antigen (Fig. 2a). The magnitude of antibody boosting to individual antigens was variable, with only two infants demonstrating IgG rises to all five antigens (Fig. 2c). No difference was observed in F:MR in those with or without subsequent serological evidence of exposure (Supplementary Fig. 2). IgG dynamics across the life course to GAC, SLO, SpyAD, SpyCEP and DNAseB were explored using serum at recruitment to the SpyCATS study to model age-stratified antibody distributions (*n* = 413; age range, 0–85 years). IgG levels rose rapidly during early childhood with a plateau observed for all antigens by 5 years and waning seen in older age in SLO-specific, SpyAD-specific and DNAseB-specific IgG (Fig. 2d). Strong positive correlations were seen among all five antibody responses, with coefficients ranging from 0.65 to 0.86 (*P* < 0.0001 for all comparisons; Supplementary Fig. 3).

### Serological responses to culture-confirmed *S. pyogenes* events

**Fig. 3:**
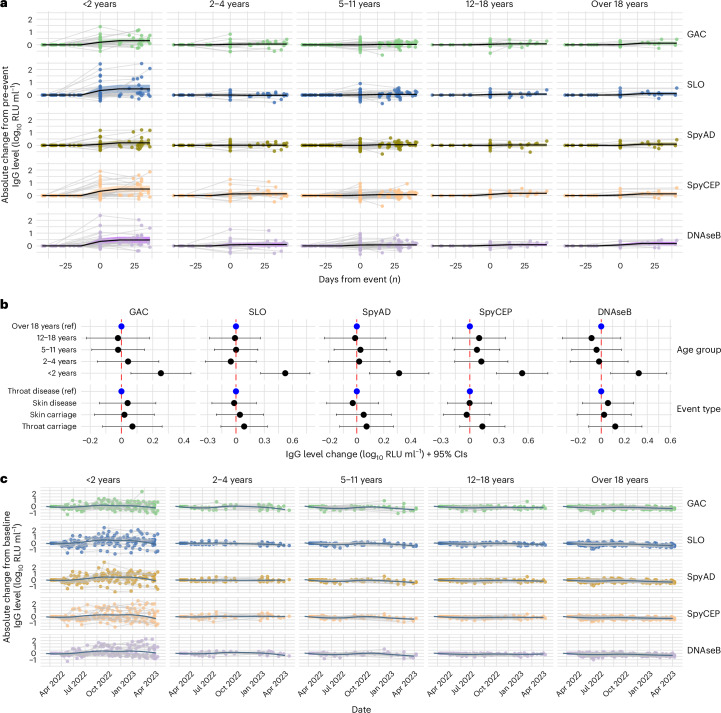
Blood IgG antibody profiles around culture-confirmed *S. pyogenes* events. **a**, Individual IgG antibody profiles by age group around microbiologically confirmed *S. pyoge*nes events, where pre-event titers and at least one subsequent titer were measured (*n* = 163 events). IgG was normalized to pre-event levels. Each dot represents an individual IgG level relative to the baseline titer. Gray lines connect individual participants’ IgG measured before, during and after events. Solid black lines represent the geometric mean log_10_-transformed IgG level changes across participants, grouped by temporal relationship to the event. Shaded areas around the lines represent 95% CIs. **b**, Forest plot showing the association between age group and event type with absolute IgG level changes around culture-conformed events (*n* = 150 events). Points represent estimated group differences, with horizontal bars indicating 95% CIs derived from mixed-effects linear regression models. **c**, Longitudinal blood IgG profiles in participants (*n* = 290) without microbiologically confirmed *S. pyogenes* events during the study period. IgG levels were normalized to individual participants’ baseline titers. Each dot represents an individual antibody titer relative to baseline, and gray lines connect titers measured over time. Dark blue lines represent geometric mean IgG level change from baseline by month, smoothed with the LOESS method. LOESS, locally weighted scatterplot smoothing; ref, reference.

**Extended Data Fig. 1:**
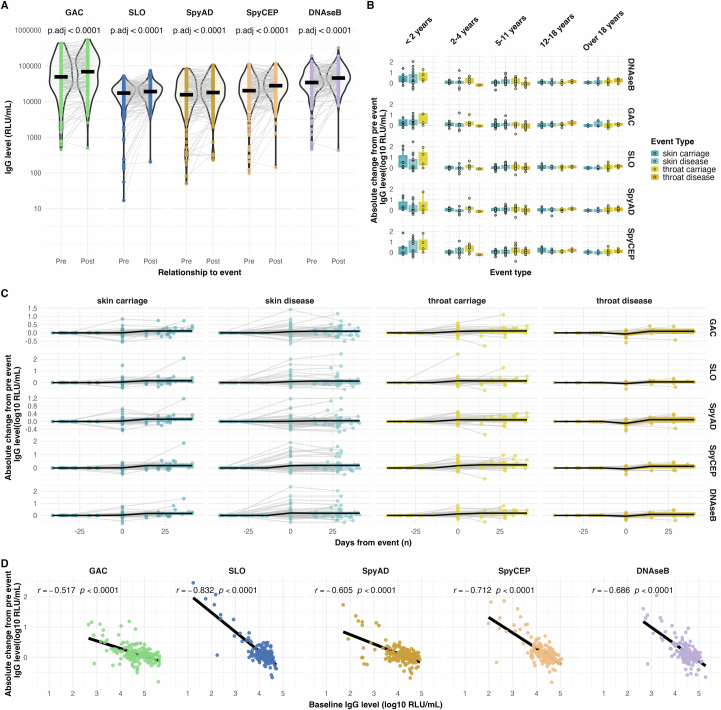
Blood IgG antibody profiles around culture-confirmed *S. pyogenes* events. **a**) Blood IgG antibody levels around microbiologically confirmed events. IgG levels from paired samples before and after a microbiologically confirmed event in the SpyCATS study (n = 150) were measured. IgG levels were compared using a paired Wilcoxon signed-rank test. Median IgG level is represented by centre line. P-values were adjusted for multiple testing using the false discovery rate (FDR) correction. **b**) Absolute Blood IgG increases around culture confirmed events by event type. P values are derived from Kruskall-Wallis tests to compare absolute IgG increases between multiple event types. No significant differences between groups were observed. Box plots show medians, IQR, 1.5×IQR whiskers and outliers. **c**) Individual IgG antibody profiles by age group around microbiologically confirmed *S. pyogenes* events, where pre-event IgG levels and at least one subsequent IgG level were measured (n = 163 events). IgG was normalized to pre-event levels. Each dot represents an individual IgG level relative to the baseline level. Grey lines connect individual participants’ IgG measured before, during and after events. Solid black lines represent the mean log_10_ transformed IgG level changes across participants, grouped by temporal relationship to the event. Shaded areas around the lines represent 95% confidence intervals calculated using the mean and standard error of the log-transformed IgG levels. **d**) Association between baseline (pre-event) IgG level and absolute increase in IgG level between pre and post event (n = 150 events). Pearson’s correlation coefficient (*r*) was calculated for each antige, using a two-sided test without correction for multiple comparisons.

The kinetics of IgG levels to GAC, SLO, SpyAD, SpyCEP and DNAseB before and after culture-confirmed *S. pyogenes* events were explored using serially collected dried blood spots (DBSs). Paired pre-event and post-event DBS samples were available for 150 events (13 pharyngitis, 67 pyoderma, 1 mixed disease, 34 pharyngeal carriage, 35 skin carriage; Supplementary Table 1). The median time between measurement was 70 days (IQR, 59–95), with no significant difference in timing between disease and carriage events. Significant rises in IgG levels to all five antigens were seen after events, ranging from 0.10 to 0.19 log_10_ RLU ml^−1^ (*P* < 0.0001 for all comparisons; Extended Data Fig. 1a) although with substantial heterogeneity. Younger participants, particularly those younger than 2 years of age, had higher IgG rises (Fig. 3a,b), with responses after all event types (Extended Data Fig. 1b,c). In a generalized linear mixed-effects model accounting for age and event type, participants younger than 2 years of age had significantly greater absolute increases in IgG levels, ranging from 0.25 to 0.54 log_10_ RLU ml^−1^, compared to adults (GAC *P* = 0.011, SLO *P* = 0.00017, SpyAD *P* = 0.0068, SpyCEP *P* = 0.00013, DNAseB *P* = 0.013; Fig. 3b). Baseline IgG levels and absolute increases after events were inversely correlated (coefficients −0.5 to −0.83, *P* < 0.0001 for all antigens; Extended Data Fig. 1d). Rises in IgG were equivalent after pyoderma, and asymptomatic pharyngeal and skin carriage, when compared to pharyngitis (Fig. 3b and Extended Data Fig. 1c). In individuals with no culture-confirmed *S. pyogenes* during the study, IgG levels remained stable over the 13-month period, other than in infants younger than 2 years of age where several individuals had IgG rises and others demonstrated waning from baseline levels (Fig. 3c).

### Antigen-specific IgG and protection from culture-confirmed events

**Fig. 4:**
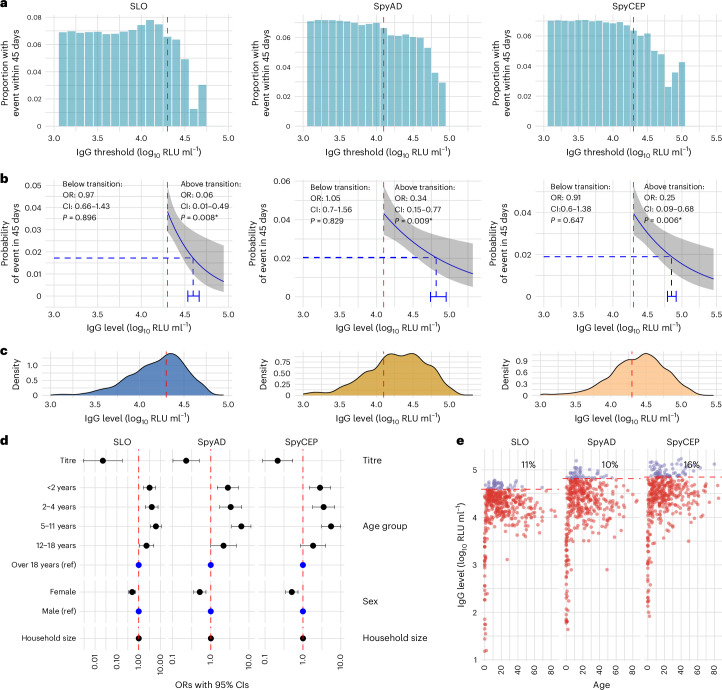
Association between IgG levels to conserved vaccine antigens and protection from culture-confirmed *S. pyogenes* events. **a**, The proportion of visits (*n* = 4,677) with IgG levels above each threshold associated with a culture-confirmed *S. pyogene*s event within 45 days. IgG levels were measured from *n* = 1,987 visits and assumed to remain constant between measurements. **b**, Piecewise logistic regression analysis with mixed effects to explore the relationship between IgG level and event within 45 days (*n* = 4,677 visits with 1,987 antibody measurements). Transition points in the relationship between IgG levels and event risk were identified from **a** and refined using AIC analysis. Piecewise logistic regression with titers above and below the transition point was performed. The blue line shows the fitted regression model, capturing the association between titer levels above the breakpoint and the probability of a *S. pyogenes* event within 45 days; gray shading represents the 95% CIs for model predictions. OR for each log_10_ IgG level change compared to the transition point, 95% CIs and *P* values from two-sided piecewise logistic regression models are displayed. No multiple testing correction was applied. The red vertical line indicates the transition point. Putative 50% protective thresholds were calculated using 10-fold cross-validation as the IgG level at which the predicted probability of an event with 45 days was 50% that of the predicted probability at the transition point. 50% thresholds are plotted with blue dashed lines along with 95% CIs (blue error bars) (*P < 0.05). **c**, Density plot showing the distribution of IgG measurements (*n* = 1,987) in relation to IgG level. The red line marks the transition identified in **a**. **d**, Forest plot to visualize the association between IgG level above the transition point for each conserved antigen (*n* = 4,677 visits with 1,987 antibody measurements) and any culture-confirmed event within 45 days. Points represent OR estimates from a mixed-effects logistic regression model adjusting for age, sex and household size. Horizontal bars represent 95% Wald CI. **e**, IgG level distribution by age in years at study baseline (*n* = 413), including the percentage of participants with IgG levels above (purple) the identified 50% protective threshold (red dashed line). ref, reference.

**Extended Data Fig. 2:**
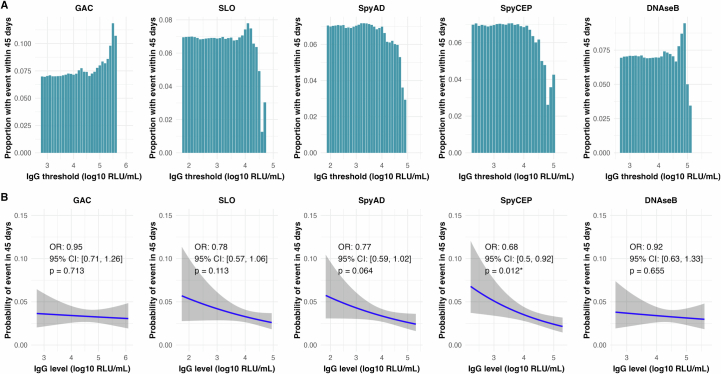
Association between IgG levels to conserved antigens and protection from culture confirmed *S. pyogenes* events. (**a**)The proportion of visits (n = 4677) with IgG levels above each threshold with a culture-confirmed *S. pyogenes* event within 45 days. IgG levels were measured from n = 1,987 visits and assumed to remain constant between measurement. (**b**) Logistic regression analysis with mixed effects to explore the relationship between IgG level and event within 45 days. The blue line shows the fitted regression model grey shading represents the 95% confidence intervals for model predictions. Odds ratios per log_10_ IgG level increase, 95% confidence intervals, and p-values for the logistic regression model are displayed.

**Extended Data Fig. 3:**
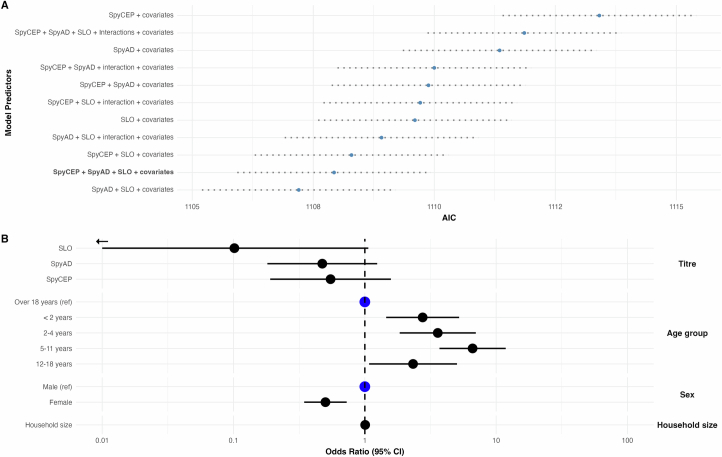
Exploring synergistic effects of anti-SLO, -SpyAD and -SpyCEP IgG with protection from culture confirmed events. (**a**) IgG levels (above the transition point of 4.3 log10 RLU ml−1 for SLO, 4.1 log10 RLU ml−1 for SpyAD and 4.3 log10 RLU ml−1 for SpyCEP) were included in all possible combinations with and without interaction terms and including covariates known to alter risk of *S. pyogene*s events: age group, sex and household size. AIC value for each model is plotted +− 2 represented by dotted lines. This allowed the selection of the model including IgG levels to SLO, SpyAD and SpyCEP without interaction terms (in bold text) to be used to explore synergistic effects between antigens. (**b**) Forest plot to visualise the association between IgG level above transition point for each conserved antigen and any culture-confirmed event within 45 days (n = 4677 visits with 1987 antibody measurements). Odds ratios (points) were calculated from a mixed effects logistic regression model adjusting for factors known to alter risk of *S. pyogene*s events: age, sex and household size. Horizontal bars represent 95% Wald confidence intervals. AIC = Akaike information criterion.

IgG to conserved *S. pyogenes* vaccine antigens from 1,987 timepoints in 431 participants was used to explore protection against 196 culture-confirmed events (Supplementary Fig. 1b). This included measurements at baseline, before, during and after events in cases and before and after events in household contacts where no *S. pyogenes* culture-confirmed event was detected at the time of an index event. At each IgG threshold, the proportion of visits with *S. pyogenes* events in the subsequent 45 days was determined (Extended Data Fig. 2a), and mixed-effects logistic regression models were used to establish the association between *S. pyogenes*-specific IgG levels and the probability of subsequent *S. pyogenes* events. With this model, only higher anti-SpyCEP IgG was associated with reduced odds of *S. pyogenes* events (odds ratio (OR) per log_10_ IgG increase 0.68, 95% confidence interval (CI) 0.5–0.92, *P* = 0.012; Extended Data Fig. 2b). For vaccine antigens SLO, SpyAD and SpyCEP, but not GAC or DNAseB, the relationship between IgG levels and probability of events appeared nonlinear, characterized by a plateau effect at lower antibody levels, followed by a downward slope (Fig. 4a and Extended Data Fig. 2b). We, therefore, employed piecewise regression to model the distinct portions of the relationship for SLO, SpyAD and SpyCEP. Transition points between portions were determined visually (Fig. 4a) and confirmed with iterative point increments and assessment of Akaike information criterion (AIC), where values within 2 were considered similar. Above the transition point, a significant reduction in culture-confirmed events within 45 days was seen for SLO (OR per log_10_ increase above transition point 0.06, CI 0.01–0.49, *P* = 0.008), SpyAD (OR 0.34, CI 0.15–0.77, *P* = 0.009) and SpyCEP (OR 0.25, CI 0.09–0.68, *P* = 0.006) (Fig. 4b). The majority of all IgG levels measured were above the transition points (Fig. 4c). IgG levels to all three antigens remained associated with protection in models adjusting for age, sex and household size (Fig. 4d). To explore potential synergistic effects between IgG responses to SLO, SpyCEP and SpyAD, we fitted models including all IgG levels above the identified transition points. Although the protective trends for each antigen were retained, no single antigen emerged as a significant or dominant predictor, and combining responses offered limited improvements in model performance (Extended Data Fig. 3).

To obtain putative 50% protective thresholds for SLO-IgG, SpyAD-IgG and SpyCEP-IgG, we implemented a 10-fold cross-validated piecewise mixed-effects logistic regression, identifying the IgG level at which the probability of an event reached 50% of the maximum predicted probability above the transition point (Fig. 4b). These thresholds were 39,209 (CI 34,037–45,904) RLU ml^−1^ for SLO, 65,454 (CI 54,748–89,448) RLU ml^−1^ for SpyAD and 70,643 (CI 61,509–82,577) RLU ml^−1^ for SpyCEP. At baseline, antibody levels from the cohort showed that 44 individuals (11%) for SLO, 43 (10%) for SpyAD and 66 (16%) for SpyCEP had IgG levels above the protective thresholds (Fig. 4e). To assess whether IgG responses to multiple antigens were associated with increased protection, we analyzed a composite variable representing the number of antigens above the putative protective threshold per timepoint. Compared to individuals with high IgG to a single antigen, those with titers above the 50% protective threshold for two or more antigens showed a trend toward reduced risk of events (OR 0.32, CI 0.07–1.46, *P* = 0.14). A statistically significant association was observed when using a more permissive 33% protective threshold; individuals with titers above this threshold for two or more antigens had reduced odds of infection compared to those with only one (OR 0.47, CI 0.25–0.86, *P* = 0.01). These findings suggest potential additive effects of conserved antigen-specific IgG at lower individual titers (Extended Data Table 1).

To confirm our findings using an orthogonal approach, an Anderson–Gill extension of Cox proportional hazards model was used, as described previously to establish epidemiological risk factors for *S. pyogenes* in this study^10^. IgG levels above the transition point were added as time-dependant covariates, accounting for clustering within households and repeated events in individuals, with adjustment for age group, sex and household size. Higher IgG levels to SLO (hazard ratio (HR) 0.04, CI 0.01–0.23, *P* = 0.00036), SpyAD (HR 0.29, CI 0.16–0.53, *P* < 0.0001) and SpyCEP (HR 0.38, CI 0.16–0.90, *P* = 0.027) were associated with protection from any culture-confirmed event (Extended Data Table 2). In models stratified by event type, protection against *S. pyogenes* carriage events was associated with higher IgG to SLO (HR 0.01, CI 0.00–0.13, *P* = 0.00012), SpyAD (HR 0.18, CI 0.08–0.41, *P* < 0.0001) and SpyCEP (HR 0.20, CI 0.09–0.49, *P* = 0.00037) (Extended Data Table 2), which was driven by protection from pharyngeal but not skin carriage (Extended Data Table 3).

### Serotype-specific anti-M IgG responses

**Fig. 5:**
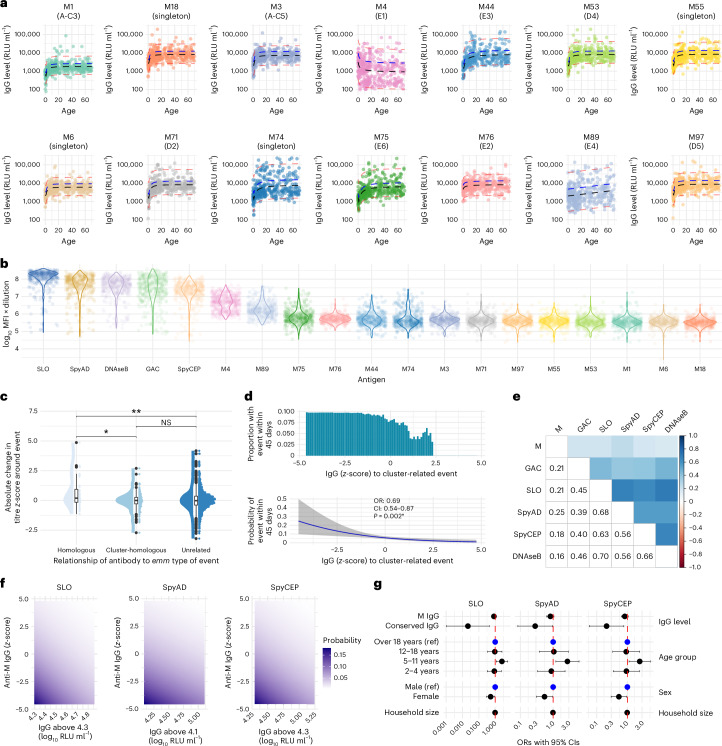
Exploring the role of type-specific anti-M protein antibodies in protective immunity. **a**, Cross-sectional profiles of anti-M IgG *z*-scores at baseline in participants without events (*n* = 402), stratified by age in years. **b**, MFIs (adjusted for dilution) for each M peptide at baseline, showing relative IgG abundance across peptides. **c**, Absolute change in anti-M IgG *z*-scores around 130 culture-confirmed, M/*emm* typed *S. pyogenes* events. Paired measurements were categorized as homologous (*n* = 40), cluster-homologous (*n* = 202) or unrelated (*n* = 1,757) to the *emm* type of the event. Box plots show medians, IQRs and 1.5× IQR whiskers. Kruskal–Wallis and post hoc Dunn’s tests with Bonferroni correction were used for comparison (*P < 0.05 and **P < 0.01). **d**, Logistic mixed-effects models were used to assess the association between M/*emm* cluster-related anti-M IgG *z*-score and the odds of an event within 45 days. Anti-M IgG (*z*-scores) before, during and after culture-confirmed M/*emm* typed events in both cases (*n* = 378 measurements, 143 events, 103 participants) and household controls (*n* = 1,366 measurements from 293 participants) were identified against the *emm* cluster-related M peptide to the M/*emm* type of the event. The cluster-related IgG level was assigned hierarchically with the homologous titer included where available; otherwise, the *emm* cluster homologous titer was selected. OR for each *z*-score change, 95% CIs and *P* values from two-sided logistic regression models are displayed (*P < 0.05). **e**, Spearman’s correlation between *z*-score normalized anti-M IgG and conserved antigen IgG levels, based on 1,651 paired measurements. **f**, Tile plot showing predicted probabilities of an event within 45 days by anti-M IgG (*z*-score) and conserved antigen IgG above the transition point. Logistic mixed-effects models included both antibody types. **g**, Forest plot from fully adjusted logistic mixed-effects models (*n* = 1,652 samples from 307 individuals), showing ORs (points) and 95% Wald CIs (horizontal lines) for anti-M IgG and conserved antigen IgG above the transition point. Models were adjusted for age, sex and household size. NS, not significant; ref, reference.

To compare anti-M humoral immunity with conserved antigen-specific IgG, antibody levels to a range of M peptides were measured. Although over 275 *emm* types exist, several M/*emm* clusters have been defined based on M protein structural similarities. In vitro cross-reactivity (and potentially cross-protection) may exist within each cluster^15,30,31^. *S. pyogenes* isolates from published studies in The Gambia show a wide diversity with multiple different *emm* types and no single *emm* type predominance^32–34^. *emm* type distribution from this study is described elsewhere^10,32^. In baseline cohort sera, IgG levels to 14 M/*emm ‘*cluster-representative’ 50-mer hypervariable region M peptides demonstrated more variability across the life course than for conserved antigen-specific IgG (Fig. 5a and Supplementary Fig. 4)^21^. Raw median fluorescence intensity (MFI) antibody levels (unadjusted to levels in standard material) across all antigens showed a hierarchy of signal ranging from high SLO, SpyAD, SpyCEP, GAC and DNAse B to moderate levels of anti-M4, anti-M89 and anti-M75 IgG and to lower or heterogenous levels of other M-specific antibodies (Fig. 5b). At birth, IgG to all M peptides was significantly lower in cord serum than paired serum from mothers (Supplementary Fig. 5).

Each culture-confirmed *S. pyogenes* event was *emm* typed as previously reported^10,32^, allowing antibody data in relation to events to be categorized as homologous (M peptide matching the event M/*emm* type), cluster-homologous (non-matching M peptide in same *emm* cluster as event M/*emm* type) or unrelated. Antibody levels were measured in paired pre/post samples to 14 cluster-representative M peptides and to five additional M peptides from the E3 *emm* cluster, which contains *emm* types commonly seen in The Gambia (*n* = 1,999 paired measurements from 130 *emm* typed events). Anti-M IgG RLU ml^−1^ values were *z*-score transformed to allow aggregation and comparison across *emm*/M types. Absolute *z*-score increases before and after events were categorized into homologous (M peptide matching the event *emm* type, *n* = 40), cluster-homologous (non-matching M peptide in same cluster as event *emm* type, *n* = 202) or unrelated (*n* = 1,757) responses. Absolute increases in IgG levels induced by *S. pyogenes* events were greater for homologous events than for cluster-homologous (*P* = 0.016) and unrelated (*P* = 0.0074) events (Fig. 5c).

### M serotype-specific IgG and protection

We next explored whether anti-M IgG was associated with protection. For each *emm* typed event (*n* = 143), the homologous or, if unavailable, the cluster-homologous anti-M IgG *z*-score (hereafter called cluster-related) was identified in cases before, during and after the event and in household contacts before and after the event. In total, 1,652 measurements from 1,215 timepoints and 307 individuals were included. For comparison, a mean *z*-score of event-unrelated anti-M IgG was also generated for each timepoint. Cluster-related IgG was correlated with mean unrelated anti-M IgG (correlation coefficient 0.59, *P* < 0.0001).

In a mixed-effects logistic regression model, higher cluster-related anti-M IgG was associated with lower odds of any culture-confirmed event (OR 0.69 per unit *z*-score, CI 0.54–0.87, *P* = 0.002; Fig. 5d), which was confirmed in a model adjusted for age, sex and household size (OR 0.74, CI 0.58–0.95, *P* = 0.019; Extended Data Table 4). Piecewise regression models for anti-M *z*-scores demonstrated higher AIC scores. Furthermore, models replacing cluster-related IgG with mean event-unrelated M IgG also demonstrated protective association of anti-M IgG (OR 0.69, CI 0.53–0.89, *P* = 0.005) but explained the data less well (AIC 813 versus 800 for models with matched anti-M IgG).

**Extended Data Fig. 4:**
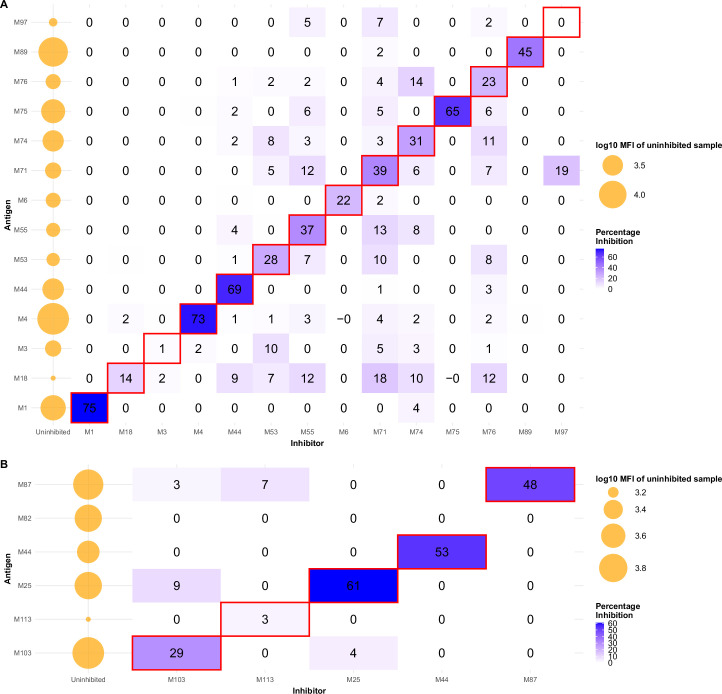
Specificity of multiplex assays to measure anti-M IgG. (**a**) Specificity of the 14-plex assay to measure IgG to M/emm cluster representative M peptides. (**b**) Specificity of 6-plex assay to measure IgG to E3 M/*emm* cluster M peptides. Positive material consisting of intravenous immunoglobulin supplemented with pooled human serum was incubated with M-peptide inhibitors at a concentration of 2 µg/mL, prior to proceeding with a standard 14-plex, or 6-plex, assay to measure IgG to M/*emm* cluster representative M peptides. Percentage MFI inhibition in relation to the MFI of uninhibited material was calculated as ((MFI Control − MFI Inhibited sample)/MFI control) × 100. Yellow circles represent the magnitude of the MFI of the inhibited signal for each antigen (as a surrogate marker of overall concentration of each antibody in immunoglobulin supplemented with pooled human serum). MFI = Median fluorescent intensity. Numbers in each cell is the percentage inhibition. Red boxes indicate homologous inhibition.

**Extended Data Fig. 5:**
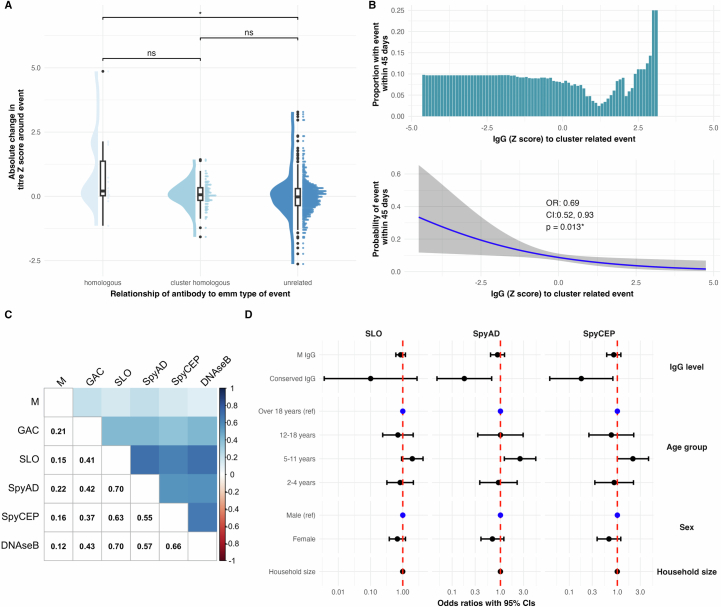
Sensitivity analysis exploring the role of type specific anti-M protein antibodies in protective immunity. (**a**) Absolute change in anti-M IgG Z-scores around 130 culture-confirmed, M/emm-typed *S.* *pyogenes* events. Only IgG measurements to M peptides with >45% homologous inhibition in inhibition assays (M1, M4, M44, M75, M89, M25, M87) were included. Paired measurements were categorised as homologous (n = 15), cluster-homologous (n = 104), or unrelated (n = 605) to the M/emm type of the event. Box plots show medians, IQRs, and 1.5×IQR whiskers. Comparisons were made using the Kruskal–Wallis test followed by Dunn’s test with Bonferroni correction (P < 0.05). (**b**) Logistic mixed effects models were used to assess the association between M/*emm* cluster-related anti-M IgG Z-score and the odds of an event within 45 days. Anti-M IgG (Z scores) before, during and after culture-confirmed M/*emm*-typed events in both cases (n = 247 measurements, 92 events, 74 participants) and controls (n = 791 measurements, 246 participants). Cluster-related titres were hierarchically assigned: homologous if available, otherwise cluster homologous. (**c**) Spearman’s correlation between anti-M IgG Z-scores and conserved antigen IgG levels from 969 paired measurements. (**d**) Forest plot from fully adjusted mixed effects logistic regression models including anti-M IgG (Z-score), conserved antigen IgG (above transition point), age group, sex, and household size. 969 measurements from 859 timepoints and 263 individuals were included. Points represent odds ratios and horizontal bars show 95% Wald confidence intervals. OR = Odds ratio; CI = confidence interval; RLU = Relative Luminex Unit.

Unlike the strong correlation and collinearity between conserved antigen IgG levels (Supplementary Fig. 3), correlation between cluster-related anti-M IgG and each conserved antigen IgG level was low (coefficients 0.16–0.25; Fig. 5e). We, therefore, sought to establish the relative contribution of conserved and anti-M IgG to protection, using AIC criteria to identify the best-fitting model and exclude significant interactions between IgG to M and conserved antigens. IgG levels above the transition point for SLO, SpyAD and SpyCEP were included in mixed-effects logistic regression models, along with cluster-related anti-M IgG *z*-score, age, sex and household size. Anti-SLO (OR 0.02, 95% CI 0.00–0.44, *P* = 0.013), anti-SpyAD (OR 0.28, 95% CI 0.08–0.95, *P* = 0.041) and anti-SpyCEP (OR 0.21, 95% CI 0.06–0.77, *P* = 0.0018) were independently associated with protection in each model (Fig. 5f,g). An independent but non-significant trend toward protection with cluster-related anti-M IgG was also observed. Of note, the specificity of some anti-M assays was limited, as assessed by competitive inhibition, likely in part due to low MFI in IVIG derived from HICs. Despite optimizing this signal as best possible with new pooled standards, specificity remained low for some anti-M assays (Extended Data Fig. 4). Sensitivity analyses using only seven M peptides with the best specificity demonstrated consistent findings across all M-related analyses (Extended Data Fig. 5).

### Functional immunoassays

To explore the relationship between binding IgG levels and functionality, a subsample of 114 sera was randomly selected within IgG strata to represent a wide range of antibody levels. Assays were used to measure the ability of sera to inhibit SLO-mediated hemolysis of erythrocytes^35^, SpyCEP-mediated interleukin-8 (IL-8) cleavage^36^ and potentiation of THP-1 cell phagocytosis of SpyAD-bound beads. Opsonophagocytic activity against GAC-bound beads and whole M1 *S. pyogenes* was also assessed^37^.

**Fig. 6:**
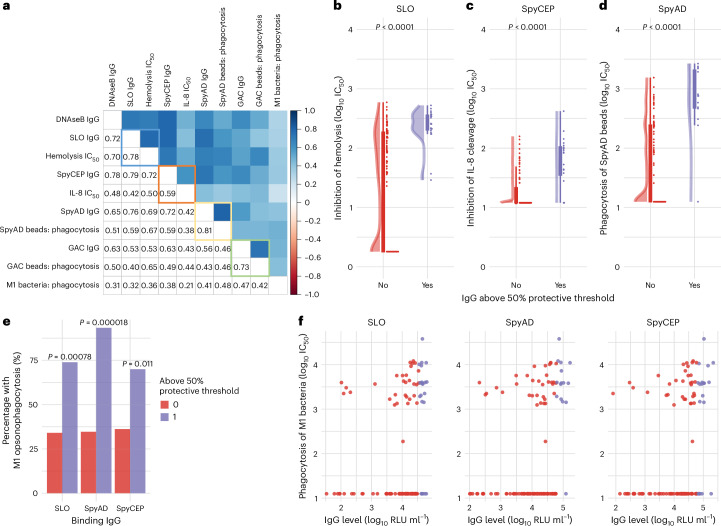
Association between protective IgG profiles and in vitro inhibition of function and promotion of opsonophagocytosis. **a**, Correlation coefficients (Spearman’s method) between binding IgG titers and functional immunoassays in *n* = 114 serum samples randomly selected across a broad range of binding IgG levels. Highlighted squares indicate the specific relationship between binding titers and the immunoassay that directly measures the function of the corresponding antigen. Blue square represents SLO, orange represents SpyCEP, yellow represents SpyAD and green represents GAC. **b**–**d**, Relationship between binding IgG levels and functional activity in serum samples (*n* = 114): IgG binding levels to SLO and inhibition of SLO-mediated hemolysis (*n* =114) (**b**), IgG binding levels to SpyCEP and inhibition of SpyCEP-mediated IL-8 cleavage (*n* = 114) (**c**) and IgG binding levels to SpyAD and promotion of phagocytosis of SpyAD-coated beads by THP-1 cells (*n* = 114) (**d**). Box plots show the IQR (box) and 1.5× IQR whiskers. Points beyond whiskers represent outliers. IC_50_ values between those above and those below 50% protective thresholds were compared with two-sided Wilcoxon tests. **e**, Proportion of participants with titers above and below 50% protective thresholds with any detectable opsonophagocytosis of M1 bacteria. Proportions between groups were compared with a two-sided Fisherʼs exact test. **f**, Relationship between IgG binding titers to SLO, SpyAD, SpyCEP and opsonophagocytosis of M1 bacteria by THP-1 cells. Binding IgG level above (purple) and below (red) the 50% protective threshold is demonstrated.

Positive correlations between binding IgG levels and functional activity were strongest for anti-SpyAD (0.81, *P* < 0.0001), followed by SLO (0.78, *P* < 0.0001) and GAC (0.73, *P* < 0.0001), with a modest correlation between anti-SpyCEP IgG levels and inhibition of IL-8 cleavage activity (0.59, *P* < 0.0001) (Fig. 6a). Sera with binding IgG levels above putative 50% protective thresholds demonstrated significantly higher functional activity against SLO (*P* < 0.0001; Fig. 6b) and SpyCEP (*P* < 0.0001; Fig. 6c) and significantly higher opsonophagocytosis of SpyAD-coated beads (*P* < 0.0001; Fig. 6d). Opsonophagocytosis of M1 bacteria was observed in a greater proportion of samples above the 50% protective threshold compared to below it for SLO (70% versus 36%, *P* = 0.011), SpyAD (92% versus 36%, *P* = 0.00012) and SpyCEP (72% versus 36%, *P* = 0.0080) (Fig. 6e). Modest but statistically significant correlations were seen between binding IgG levels to SLO (0.32, *P* < 0.00044), SpyAD (0.41, *P* < 0.0001), SpyCEP (0.38, *P* < 0.0001) and opsonophagocytic activity against whole M1 bacteria (Fig. 6a,f).

## Discussion

In a high-burden setting for *S. pyogenes* disease, we demonstrate that serological profiles are driven by intense exposure in the first years of life. We also demonstrate, to our knowledge for the first time, that high IgG levels to SLO, SpyCEP and SpyAD are associated with protection from culture-confirmed events, independent of type-specific antibodies. Notably, these conserved antigens are included in several *S. pyogenes* vaccines in development^3,22,24,26,38^.

After waning of maternal IgG, we observed a rapid rise and plateauing of serum IgG levels in the first few years of life and gradual waning of IgG to some antigens in older adults, similar to findings from Fiji and Uganda^39,40^. This likely reflects heavy exposure to *S. pyogenes* in this environment, in keeping with the high incidence rates of 409 per 1,000 person-years for carriage and 542 per 1,000 person-years for disease in children younger than 5 years of age in The Gambia^10^. The most vigorous responses to events occurred in children younger than 2 years of age, regardless of detection site or presence of symptoms. This is most likely explained by the observation that greater absolute IgG rises after exposure are seen in those with low pre-event levels (consistent with prior observational and human challenge studies^25,41,42^), which, in turn, are found most commonly in young infants. Immune responses to *S. pyogenes* pharyngitis have been extensively characterized, yet our data demonstrate that pharyngeal carriage, skin disease and skin carriage are equally important drivers of early-life immunological responses^23,25,43,44^. Interestingly, adults with culture-confirmed *S. pyogenes* disease or carriage demonstrated limited boosting of IgG to conserved antigens, highlighting the limitations of using streptococcal serology to provide evidence of recent infection in high-burden settings. It is possible that IgG boosting may be greater during toxin-mediated or invasive *S. pyogenes* infections and in the context of acute rheumatic fever (ARF), which were not explored in our study^45^. Interestingly, although placental transfer of IgG to conserved protein antigens was complete, transfer of IgG to GAC and M peptides was significantly lower. This difference may be attributable to differences in IgG subclass distribution or other antibody characteristics, such as glycosylation or inflammatory profile, that influence placental transport efficiency^46–48^.

Understanding the extent of early-life exposure is important in determining the age at which *S. pyogenes* vaccines should be introduced. We observed that 23% of infants have experienced a likely serological priming event by 11 months, yet only 2% of participants younger than 2 years of age had baseline IgG levels above our putative 50% protective threshold for each antigen. Recent modeling assuming protection in line with World Health Organization preferred product characteristics suggested that introduction of an *S. pyogenes* vaccine in 5-year-old children would have greater impact than vaccinating at birth^49^. However, data from New Zealand suggest that exposure to diverse streptococcal genotypes alongside heightened serological responses in early life may be important priming events for ARF and RHD^43,45^. The intense serological activity that we observed in early life may suggest that pathological immune priming in susceptible individuals begins before the age of 5 years. Enhanced protection earlier in life could be critical in preventing RHD in high-burden settings. Future vaccine trials and modeling should evaluate the impact of vaccinating children under the age of 2 years. On the other hand, we observed substantial heterogeneity in IgG levels to conserved vaccine antigens even in adolescence and adulthood. Given that only a minority of participants demonstrated levels above our putative 50% protective thresholds, a successful vaccine could boost protective responses even in older children. As the assays described will be used in clinical evaluation of a leading *S. pyogenes* vaccine, our study provides valuable data against which early-phase vaccine trial immunogenicity can be compared. It is crucial, however, that our data are calibrated to international *S. pyogenes* antibody standards when developed in the future, allowing wider comparison and validation.

Future vaccine trial endpoints will likely be *S. pyogenes* pharyngitis and skin infection, not carriage. It is important to note that the endpoints studied here were all *S. pyogenes* events, and it is plausible that the immune responses required to prevent pharyngitis and pyoderma are different than those that protect against carriage. Although our study lacked power to explore protection from specific events, especially with low pharyngitis incidence, the clearest protective signal was observed for pharyngeal carriage, despite similar numbers of skin carriage and disease events. Environmental factors such as physical trauma and hygiene may be more important in the pathogenesis of *S. pyogenes* skin events, whereas humoral immunity may play a greater role in preventing pharyngeal events^10,50^. This has implications for evaluating whether vaccine-induced immunity can prevent pharyngitis and pyoderma in future trials.

Although our conserved antigen IgG assay has been extensively characterized^28^, our anti-M IgG assays suffered from poor specificity for some peptides. This may be due to a combination of anti-M cross-reactivity and variable amounts of type-specific IgG in the IVIG/pooled serum used for assay validation. Despite limitations, we observed low and heterogenous anti-M IgG levels at study entry to multiple type-specific hypervariable region peptides, with the highest responses seen to M peptides from *S. pyogenes* types observed more frequently in The Gambia, including *emm*4, *emm*89 and *emm*75 (refs. ^32–34^). The streptococcal M protein is a major immunogen and potential vaccine target, with type-specific antibodies shown to be protective in vitro and in limited observational data^15,17–19,51,52^. In contrast to conserved streptococcal antigens, type-specific IgG dynamics are likely to be more variable in settings like The Gambia with documented high *emm* diversity, frequent *emm* type introductions into households and no strain dominance^10,32–34,53^. At present, multivalent vaccines to M protein hypervariable regions, based largely on *emm*/M types common in HICs, are the only *S. pyogenes* vaccines to have progressed to phase 2 trials^54^. The efficacy of multivalent M-based vaccines would rely on the degree of cross-protective immunity within M/*emm* clusters^21,30,31^. A protective effect of cluster-related anti-M IgG was apparent, although these analyses were likely limited by power.

Our findings that IgG levels to conserved antigens SLO, SpyAD and SpyCEP may be associated with protection, independent of anti-M immunity, are particularly pertinent to LMICs where M/*emm* type diversity is greatest^20,21^. We demonstrate a strong correlation between binding IgG and functionality, although SpyCEP-mediated IL-8 cleavage inhibition was less clearly correlated with anti-SpyCEP IgG, perhaps explained by binding of antibodies to non-enzymatic regions of SpyCEP. The association between IgG binding levels and opsonophagocytosis of *emm*1 bacteria is also likely mediated by antibodies to conserved targets, either measured or unmeasured, given low levels of anti-M1 IgG in the cohort and no prior documentation of *emm*1 bacteria in The Gambia in multiple studies^32–34^. Of note, higher IgG levels to GAC and DNAseB were not associated with protection in our data. GAC is a promising preclinical vaccine candidate contained within several leading vaccines, and vaccine-induced protection may differ from naturally acquired immunity^3,55–58^. The anti-SLO, anti-SpyAD and anti-SpyCEP IgG-associated protection that we observed may be a surrogate of unmeasured immune responses. These could include innate, T cell and memory B cell responses, targeting antigens beyond our limited panel, such as those incorporated in candidate vaccines, or other antigens yet to be characterized^3,7,59,60^. Furthermore, it is possible that cross-reactive immune responses to pathogens with some antigenic similarity to *S. pyogenes*, such as *Streptococcus dysgalactiae* subsp. *equisimilis* (especially in SLO and SpyAD^61^), may have contributed to the responses that we measured. Future vaccine trials, human challenge studies and cohorts with longer periods of follow-up and broader immunological investigation will help gain deeper understanding into generation and maintenance of protective immunity and establish whether antibodies to these vaccine antigens are true mechanistic correlates of protection^7^.

Our study has additional limitations. Cohort 1 did not undergo microbiological sampling. As such, we are unable to directly relate the increases in IgG levels to new *S. pyogenes* exposure in the first year of life in this cohort^61^. We likely missed *S. pyogenes* events in cohort 2 for several reasons. We previously demonstrated the limited sensitivity of culture compared to molecular methods in this setting^53,62^. Nonetheless, culture positivity is directly related to quantitative polymerase chain reaction (PCR) bacterial load^53,62^ and, therefore, remains a relevant, if insensitive, outcome. Second, our monthly routine sampling likely missed carriage events, given the short carriage duration of *S. pyogenes* (median 4 days) that we have reported^10^. The immunological responses observed in children younger than 2 years of age with no culture-confirmed events reflects this. Although we have detailed representation in the early years of life in our cohorts, we have fewer older adults in the study and, therefore, did not have the capacity to assess impact of antibody titer waning on susceptibility to infection in older age. The framework used to select timepoints for antibody measurement and assessing protection, rather than unbiased universal testing of all samples, may have introduced biases. We mitigate this by demonstrating that IgG levels in older participants without culture-confirmed events remained broadly constant and by testing every timepoint in participants under the age of 2 years. Furthermore, we tested all samples from culture-confirmed cases and household contacts longitudinally around events, where antibody levels were most likely to change. Finally, the study did not have sufficient power to determine protective thresholds for disease and carriage separately, which will be a key consideration for defining correlates of protection in future vaccine studies with disease-specific endpoints.

In conclusion, our study represents a unique resolution of immunological sampling from intensively followed cohorts across the life course in a high-burden setting for *S. pyogenes* and RHD, using robust and reproducible immunoassays^28,35–37^, focusing on antigens within leading candidate vaccines^3^. We demonstrate the dynamic evolution of humoral immune responses in children younger than 2 years of age, a previously underrepresented group in observational studies. Our data suggest that antibodies to both conserved vaccine antigens and M peptides may be associated with protection from culture-confirmed *S. pyogenes* events, providing optimism for both conserved and multivalent M protein approaches. Additional well-designed epidemiological and multiphase clinical vaccine trials in high-burden settings are urgently required to further understand mechanisms of protective immunity and to identify a tractable correlate of protection.

## Methods

### Study participants and sampling

#### Mother–child pairs during the first year of life

Mother–child pairs from an urban clinic, The Gambia, participating in a trial of maternal immunization with MenAfriVAC were included (NCT03746665). All samples were obtained from consenting mother–child pairs where the mother had been vaccinated with meningococcal serogroup A conjugate vaccine between 28 weeksʼ and 34 weeksʼ gestation. Participants were vaccinated between December 2018 and October 2019. All participants were included where a paired serum sample from mother at delivery and neonatal cord blood was available.

#### Household longitudinal cohort study

Participants in the SpyCATS household cohort study were included^10,63^. A total of 442 participants from 44 households were recruited and visited monthly, with an open cohort approach such that new household members were able to enroll at any monthly visit. At each monthly visit, all enrolled participants were swabbed to determine the presence of Group A beta-hemolytic streptococci (GABHS) in their normal skin and throat. Unscheduled visits took place at any time when participants reported skin sores or a sore throat (disease episodes) to the study team. Suspected disease sites were swabbed for GABHS. At enrollment, a DBS was taken from all participants and a blood sample for serum separation from all participants older than 2 years. A DBS was collected from all participants during each monthly visit and at any presentation with disease.

### *S.pyogenes* event definitions from the SpyCATS longitudinal cohort study

Disease events were defined as the presence of signs or symptoms of pharyngitis or pyoderma plus a positive culture for GABHS from the disease site. Carriage events were defined as the detection of GABHS from throat or skin swabs without symptoms or signs of disease. All GABHS were considered to be *S. pyogenes*. Symptomatic pharyngitis was characterized by the presence of a sore throat (or parental reporting of pharyngitis-like symptoms in children younger than 5 years) along with observable tonsillo-pharyngeal redness during examination. Symptomatic pyoderma was characterized by one or more purulent or crusted skin lesions.

We categorized events into two distinct types: response-focused events (RFEs) and protection-focused events (PFEs). This categorization was used to investigate the role of antibodies in both responding to and protecting against *S. pyogenes* events within the SpyCATS longitudinal cohort study.

RFEs were defined to study how the immune system responds to specific instances of disease or carriage. Disease events in this category were defined as the presence of symptomatic pharyngitis or pyoderma with a corresponding positive culture for GABHS. These events were considered as new events when no related symptomatic disease events occurred in individuals within the prior 42 days. Carriage events were classified based on a positive GABHS culture in the absence of symptoms. RFE carriage events could be defined only in absence of a disease event within the preceding 42 days or the following 14 days. This definition ensured that immune responses to a carriage event in this context were not influenced by overlapping or closely timed disease events.

PFEs were defined previously and were used to analyze risk factors for incident events, excluding weekly swabs from carriage incidence analysis and allowing simultaneous characterization of disease and carriage events^10^. We, therefore, used PFEs to explore how immune responses influence the incidence of future events. Disease events in the PFE category were defined similarly to those in RFEs but with a key difference: they could be recorded as long as they occurred at least 14 days after a previous event rather than requiring a 6-week gap. Carriage events were also defined as a positive GABHS culture at a monthly visit, in the absence of symptoms, provided there was no carriage at the two consecutive previous visits or no carriage at one preceding visit and the last positive had been more than 28 days earlier or the previous swab was positive but more than 42 days earlier. However, no exclusion was made on the basis of presence of disease, allowing simultaneous disease and carriage events to be defined.

### Sample selection for IgG level measurement

For mother–child pairs, samples used were those from mothers at delivery, neonatal cord blood at delivery and samples from infants taken at 6 months and from between 9 months and 11 months of age. From the SpyCATS cohort, a baseline sample of serum (or DBS in children younger than 2 years) was selected from all participants. DBS samples selected around RFEs were taken from the closest sample 14 days prior to the event (pre-event), the time of the event (event) and the closest sample at least 14 days after the event (post-event) as well as 3 months and 6 months after the event where available. To describe IgG level changes around events, the absolute difference in log_10_-transformed titers between the pre-event and post-event sample were used. We identified a control group of exposed, uninfected household contacts. These individuals were in the same household as an index case, were sampled within 14 days of the index event, had no *S. pyogenes* event within 90 days of the index case and had a DBS sample taken on the day of the household event or within the 28 days prior. The control group also had a sample selected for testing between 14 days and 42 days from the index event where available. For the cross-sectional analysis of age-stratified IgG levels, a single measurement per participant was taken from baseline, providing there was no *S. pyogenes* disease event at the time. In the case that both serum and DBS were measured at a single timepoint, a geometric mean IgG level was taken from the two readings. Additionally, in participants younger than 2 years of age, where IgG levels were rising fastest, a DBS sample was tested from every timepoint collected in the study.

### Sample preparation

DSB cards (Whatman Protein Saver) stored at −20 °C since collection were punched with four 6-mm punches and eluted in 630 μl of elution buffer. The elution buffer consisted of phosphate buffered saline (PBS) + 0.05% Tween 20 + 0.08% sodium azide at a ratio of 1:10 whole blood to buffer. The samples were first subjected to rotation for 30 minutes at 9×*g*, followed by overnight incubation at 4 °C. They were then further processed with an additional 15-minute rotation at 300 r.p.m.

### IgG measurement in blood for conserved *S. pyogenes* antigens

Both serum and DBS samples were tested with a characterized Luminex 5-plex assay to measure IgG levels to the conserved *S. pyogenes* antigens GAC, SLO, SpyAD, SpyCEP and DNAseB^28,29^. Antigens were coupled to magnetic carboxylated microspheres using carbodiimide chemistry. For GAC, which is polysaccharide in nature, biotinylated GAC was coupled to streptavidin-coated beads. Serum or eluted DBS samples were diluted in PBS at dilutions ranging from 1:300 to 1:60,000 and incubated for 1 hour with 1,000 beads per region per well at room temperature for 60 minutes in the dark, with shaking at 750 r.p.m. After washing, 50 µl per well of R-phycoerythrin-conjugated AffiniPure goat anti-human IgG (F(ab′)_2_ fragment-specific) secondary antibody (Jackson ImmunoResearch) was applied at a 1:70 dilution in PBS and incubated for 30 minutes in the dark with shaking at 750 r.p.m. A standard curve of Privigen IVIG in three-fold dilutions from 1:990 was added to each plate alongside a single sample of pooled serum to act as a positive control. The same lot of IVIG was used throughout the study. Each plate also included two blank wells containing only PBS and microspheres. Samples were tested in single, at a starting concentration of 1:20,000, and retested at an alternative dilution if the MFI value for any antigen fell outside of limits of standard curve accuracy^28^. All serial DBS samples from the same individual were tested on the same assay plate. The geometric mean IgG level from all repeats falling within limits of standard curve accuracy was taken as the final IgG level.

### Adaptation of Luminex 5-plex assay to measure IgG in DBS samples

It was demonstrated that using Luminex multiplex assays to measure IgG titers to *S. pyogenes* antigens from DBS samples performs consistently with their measurement in serum^29^. To confirm this observation, we performed side-by-side measurement on serum and eluted DBS samples from 18 heathy adult healthcare workers. The substudy of ‘COVID-19 Humoral ImmunE RespOnses in front-line health care workersʼ (COVID HERO study) to optimize immunoassay methods for measuring humoral responses to SARS-CoV-2 and other pathogens was approved by the UK Health Research Authority (IRAS 283461, amendment ref: NSA03). DBSs were eluted overnight in PBS + 0.05% Tween 20 + 0.08% sodium azide at a ratio of 1:10 whole blood to buffer (and, therefore, assuming a ratio of serum to buffer of 1:20). log_10_-transformed RLU ml^−1^ values were compared with the Spearman method. Very strong correlation of serum with eluted DBS samples was established (Supplementary Fig. 6)^29^.

### M/*emm* type-specific IgG measurement in a multiplex assay

We further adapted the Luminex assay to measure IgG to 14 M*/emm* type-specific M peptides, selected as representative of different M/*emm* clusters^21^, along with five additional M peptides from the E3 M/*emm* cluster, which were most common in the study (Supplementary Table 2)^10^. Peptide sequences for the 50-mer M peptide N-terminal hypervariable regions, corresponding to cluster-representative peptides, were obtained commercially (ProteoGenix). Hypervariable region peptides were constructed with a biotinylated lysine amino acid added to the C terminal of the peptide. The 15 most common *emm* clusters, based on worldwide population data, were determined^21^. For each *emm* cluster, the most frequent *emm* type globally was selected as the cluster-representative M peptide. M12, from M/*emm* cluster A-C4, was not available for inclusion in the assay. Fourteen peptides were successfully manufactured and included in a cluster-representative 14-plex assay. Additionally, five M peptides, as well as the cluster-representative M44 from the E3 cluster (the most common *emm* cluster in our study)^10^, were included in a 6-plex E3 cluster-specific assay. Biotinylated peptides were conjugated to streptavidin-coupled MagPlex beads at a concentration of 5 μg per million beads^28^.

The assay was optimized to measure M protein antibodies at 1:2,500 dilution of samples. Cluster-representative 14-plex coupled beads were added to the diluted samples at 1,000 beads per region per well. The same incubation conditions were applied as described above. Standard material consisted of 25% IVIG (Gammanorm, Octagen), 25% pooled sera from *n* = 9 participants who experienced documented *emm*25, *emm*18 and *emm*113 events and 50% pooled human sera from the SpyCATS study final visit (*n* = 244). This combination was selected to enhance type-specific antibody detection for M peptides with low MFI in commercial IVIG preparations (collected in HICs). Each assay plate included a 10-step serial dilution of the standard, starting from 1:100 in PBS. An additional 6-plex assay including beads conjugated to six peptides from the E3 *emm* cluster was also employed on specific samples, under the same assay conditions as for cluster-representative M peptide IgG measurement. The cluster-representative 14-plex assay was performed on every sample selected for measurement in the study. The additional E3 6-plex assessment was performed only around events from the E3 *emm* cluster.

### Specificity of multiplex assay to measure IgG in blood to M/*emm* type-specific hypervariable region peptides

Assay specificity was determined by competitive inhibition. 25 µl of assay standard material (Privigen IVIG supplemented with pooled human sera) was incubated with 25 µl of each non-biotinylated M peptide (acting as an inhibitor) at a final concentration of 2 µg ml^−1^ in PBS. Sample and inhibitor were incubated for 1 hour before proceeding with the assay as described above. Percentage inhibition, defined as ((MFI Control − MFI Inhibited sample) / MFI control) × 100, was calculated. Homologous inhibition (inhibition of the signal from the matched peptide-coupled bead to the inhibitor) and heterologous inhibition (inhibition of the signal from the unrelated peptide-coupled beads to the inhibitor) were both defined.

### Functional immunoassays

#### Inhibition of SLO-induced hemolysis by sera

The ability of sera to inhibit SLO-induced hemolysis was assessed using a previously characterized assay^35^. Sera were serially diluted in DPBS in a two-fold series across seven steps, from a 1:10 dilution in a round-bottom 96-well plate. A 75-μl aliquot of each diluted serum sample was incubated with 75 μl of 2,400 U ml^−1^ SLO in 40 mM DTT. After a 30-minute incubation at room temperature, 50 μl of defibrinated rabbit blood, pre-washed and diluted 1:5 in DPBS, was added to each well. Control wells included IVIG (Privigen), a no-hemolysis control (red blood cells in assay buffer alone) and a maximum hemolysis control (SLO with red blood cells). The plates were then incubated for 30 minutes at 37 °C. After incubation, the plates were centrifuged at 1,000 r.p.m. for 5 minutes at room temperature, and absorbance of 100 μl of supernatant was measured at 540 nm in a flat-bottom 96-well plate. The half-maximal inhibitory concentration (IC_50_) value, representing the serum dilution required to inhibit 50% of hemolysis, was determined for each sample by plotting optical density against the log-transformed serum dilution and fitting a four-parameter logistic (4PL) curve.

#### Inhibition of SpyCEP-mediated IL-8 cleavage

The ability of sera to inhibit SpyCEP-induced cleavage of IL-8 was assessed using a previously characterized assay^36^. Sera were diluted three-fold to achieve 1:12.5 starting dilution in DPBS containing 0.5 mg ml^−1^ bovine serum albumin (BSA) (Sigma-Aldrich) and incubated with an equal volume of full-length enzymatically active SpyCEP, produced in-house at the GSK Vaccines Institute for Global Health, at a final concentration of 10 ng ml^−1^ for 5 minutes at 4 °C. Then, 50 μl of IL-8 diluted in DPBS with 0.5 mg ml^−1^ BSA was added at 20 ng ml^−1^, and plates were incubated for 2 hours at 37 °C. Controls for maximum IL-8 cleavage (SpyCEP and IL-8 without serum) and minimum IL-8 cleavage (IL-8 only) were included on each plate, alongside a control sample of IVIG (Privigen) for each experiment.

After incubation, uncleaved IL-8 levels were quantified using a sandwich ELISA with a Human IL-8 Immunoassay Kit (Life Technologies, KAC1301) according to the manufacturer’s protocol. Samples were diluted 1:20 in standard dilution buffer, and 100 μl of the diluted sample was added to the ELISA plates preloaded with 100 μl of incubation buffer. A five-point standard curve was generated using known IL-8 concentrations reconstituted in sterile water. After the addition of 50 μl of HRP-conjugated secondary antibody, plates were incubated for 2 hours at 700 r.p.m., protected from light, and washed three times with wash buffer. TMB substrate (100 μl) was added to each well, including four chromogenic blanks, and incubated for 15 minutes at room temperature in the dark. The reaction was terminated with 100 μl of stop solution.

Absorbance was measured at 450 nm, and the chromogenic blank readings were subtracted from each test condition. The optical density for each serum sample was plotted against the log-transformed dilution, and a 4PL curve was fitted using GraphPad Prism version 10 (GraphPad Software). The IC_50_ was determined as the serum dilution required to achieve an optical density halfway between the maximum and minimum IL-8 cleavage controls.

#### Measurement of phagocytosis of bacteria and antigen-labeled beads into THP-1 cells

The ability of sera to promote opsonophagocytosis of bacteria and antigen-coupled beads was investigated in a characterized assay^37^. THP-1 cells (American Type Culture Collection, TIB-202) were cultured in RPMI 1640 HEPES buffer (Thermo Fisher Scientific) supplemented with 20% FCS, 100 IU ml^−1^ penicillin and 0.1 mg ml^−1^ streptomycin and maintained at 37 °C with 5% CO_2_. For bacterial assay preparation, an M*/emm*1 *S. pyogenes* strain (051304) was cultured overnight static in Todd Hewitt broth (THB) at 37 °C with 5% CO_2_, washed and resuspended in 0.05 M (pH 9.6) carbonate buffer containing 0.1 mg ml^−1^ FITC. After a 30-minute incubation at room temperature with gentle agitation, bacteria were washed and resuspended in phagocytosis buffer (RPMI 1640 HEPES, 0.5% BSA) at an optical density at 600 nm (OD_600_) of 0.08 (~37.5 × 10^6^ bacteria per milliliter). For antigen-coated bead preparations, FITC at 0.1 mg ml^−1^ was added to M-280 Streptavidin Dynabeads (Thermo Fisher Scientific), followed by SpyAD and biotinylated GAC antigens at a concentration of 10 μg per 3 × 10^8^ beads and incubated overnight at 4 °C. Beads containing antigens were added at 37.5 × 10^6^ beads per milliliter. Serum samples were tested in three-fold dilution from 1:50 (and repeated at 1:10 if required). 20 μl of sample was added to each well, along with IgG-depleted serum (Molecular Innovations) and Privigen IVIG as controls. Bacteria or antigen-coupled beads in 20 μl were then added to each well and incubated at 37 °C for 30 minutes at 750 r.p.m. THP-1 cells were harvested, washed and resuspended in phagocytosis buffer at 7.5 million cells per milliliter. A 10-μl aliquot was added to each well and incubated for 30 minutes at 37 °C. Phagocytosis was stopped on ice, and cells were fixed with Cytofix (BD Biosciences) for 30 minutes at 4 °C. Cells were then washed, resuspended in PBS and analyzed using an Accuri flow cytometer (BD Biosciences). The geometric mean FITC intensity within the THP-1 gate was determined (Supplementary Fig. 7), and IC_50_ values were calculated by fitting a 4PL curve to the data, with the lower constraint set to the geometric mean value of wells without serum plus one s.d.

### Statistical analyses

To obtain relative IgG levels, a five-parameter logistic (5PL) curve was fitted to the blank-subtracted MFI values obtained for each standard curve point using Bio-Plex manager software. RLU ml^−1^ values for test samples were obtained by interpolating the blank-subtracted MFI values onto the 5PL curve, and then multiplying this value by the dilution factor. RLU ml^−1^ values were log_10_ transformed for statistical analysis. IC_50_ data during functional assays were produced in GraphPad Prism version 10 by fitting 4PL curves to data. In functional assay analyses, samples with an IC_50_ below the limit of quantification (LOQ) were assigned an IC_50_ value of half the LOQ as described in assay characterization^35–37^. All remaining statistical analysis was performed with R (version 4.4.0). Fractional polynomial models were applied to log_10_-transformed cross-sectional antibody data to determine the 2.5%, 50%, 80% and 97.5% centile for each antigen by age^40,64^.

After assessment of both IgG levels and absolute antibody level changes around RFEs with Q–Q plots, histograms and the Shapiro–-Wilk test, non-parametric tests were used. After additional testing for homoscedasticity by plotting residuals, the Pearson method was used to determine correlation coefficient with levels and absolute level changes within individuals, given large sample sizes. Functional immunoassay data and IgG levels to M peptides were assessed for correlation with the Spearman method. When IgG levels were compared between two groups, a Mann–Whitney *U*-test was used, corrected for testing across multiple antigens using the false discovery rate (FDR) method. For multiple comparisons between groups, Kruskall–Wallis followed by Dunn’s test with Bonferroni correction was used. Exploring the impact of age and event type on magnitude of absolute log_10_ IgG changes around events was done with a mixed-effects linear regression analysis accounting for individuals and households as random effects. *P* values (adjusted where appropriate) less than 0.05 were considered significant.

Protection associated with IgG level and culture-confirmed events (PFEs) was explored using both logistic regression models and the Anderson–Gill extension of Cox proportional hazards models^10,65^. IgG measurements included in analysis of protection from conserved antigens were from all baseline samples; all samples in participants aged younger than 2 years; samples before, during and after microbiologically confirmed *S. pyogenes* events in cases; and samples before and after a microbiologically confirmed event in household controls. IgG levels were assumed to remain constant between measurements. Mixed-effects logistic regression models were used to explore the association of IgG with an event at the next visit so long as the next visit occurred within 45 days, to account for the monthly sampling frame in the SpyCATS study. Random effects for individuals and households were used to account for repeated sampling from individuals and household structure within the study. *P* values less than 0.05 were considered significant. Models were constructed separately for each antigen given substantial collinearity between conserved antigens. To construct piecewise regression for protection mediated by anti-SLO, anti-SpyAD and anti-SpyCEP, the transition point at which proportion of events within 45 days above each IgG threshold began to diminish was identified visually for each antigen. Next, the AIC values for 0.1 log_10_ iterations of the transition point were compared to the AIC of non-piecewise logistic regression, ensuring that AIC values were at least 2 lower using piecewise regression, and to confirm the appropriate transition point. AIC values less than 2 were considered similar. In adjusted models, AIC values were used to choose the model to best explain the data and to justify the inclusion of IgG level above the transition point only. Final models selected through this method explored the association between event in the next 45 days and IgG level above the transition point, sex, age group and household size. To explore synergistic effects of antibodies demonstrated to have association with protection, we constructed models with each combination of IgG level above the identified transition points, with and without interaction terms, to select a final model with each antigen, without interaction terms and with covariates included considering AIC values less than 2 to be similar.

To establish putative 50% protective thresholds for IgG levels in blood to SLO, SpyAD and SpyCEP, the IgG level at which the probability of any event in the next 45 days was 50% compared to IgG level at the transition point was estimated using a 10-fold cross-validation approach. To assess whether simultaneous IgG responses above these thresholds to multiple antigens conferred additive protection, we derived a composite variable representing the number of antigen-specific IgG levels above their respective protective thresholds (range, 0–3) at each timepoint during follow-up. This variable was calculated using both the 50% and a more lenient 33% protective threshold definition, and its association with culture-confirmed *S. pyogenes* events was modeled using mixed-effects logistic regression. Time in the study with IgG levels above any given number (0–3) or protective thresholds was calculated using methodology described for follow-up in this study^10^.

To explore association between IgG levels to conserved antigens and protection using an orthogonal approach, the Andersen–Gill extension of the Cox proportional hazards model was used to explore the association of antibody level with incident events, as previously described^10^. Outcomes explored in this model included any incident culture-confirmed event as well as each PFE event category. Multivariable models were selected through AIC criteria assessment, including sex, age group and household size as covariates. Models accounted for both participant ID (for individual clustering allowing for recurrent event inclusion) and household ID (for household clustering).

For analysis of M/*emm* cluster protection, each culture-confirmed *S. pyogenes* event was M/*emm* typed^10^. Having identified the cases and household controls, we used the M/*emm* type of the event to allocate a cluster-related IgG *z*-score to both cases and controls in relation to each event. If a homologous M peptide measurement (matching the event M/*emm* type) was available, it was selected; otherwise, the cluster-homologous M peptide level (representing the M/*emm* cluster) was used. This measurement was defined as the cluster-related anti-M IgG *z*-score. The mean *z*-score to unrelated M peptides to each event was identified for each timepoint and compared to the cluster-related *z*-score with Pearson’s correlation. Models incorporating the composite unrelated *z*-score were compared to those incorporating the cluster-related *z*-score using AIC criteria. Cluster-related anti-M IgG from before, during and after the event in cases and before and after the event in household controls was used to assess for protection from any microbiologically confirmed event in mixed-effects logistic regression models as described above. Finally, using AIC criteria to select the best model, a mixed-effects logistic regression model was used to explore the association between any event in the next 45 days and IgG level above the transition point for conserved antigens, cluster-homologous anti-M IgG *z*-score, sex, age group and household size.

### Ethics and inclusion statement

The studies received approval from the joint ethics committee of The Gambia Government/Medical Research Council and the London School of Hygiene & Tropical Medicine Research Ethics Committee (ref: 24005 and ref: 1585). Written informed consent was obtained from adult participants as well as from parents or guardians of participants younger than 18 years of age. Additionally, children aged 12–17 years provided assent. The studies are registered on ClinicalTrials.gov (NCT05117528 and NCT03746665).

This study was conducted in close partnership with Gambian researchers and local stakeholders throughout all stages of the research process. This study was co-designed with input from the Sukuta community, including consultation with the local Alkalo (village leader), the Officer in Charge of Sukuta Health Centre and the directorate of health services from the Gambian Ministry of Health. The study’s aims were based on a comprehensive review of the existing literature and health priorities in the region and were determined to be locally relevant. Relevant local and regional literature is cited where appropriate. All members of the Gambian research team who contributed substantially to the design, implementation, data generation or interpretation are included as co-authors. The study supported capacity building by transferring multiplex immunoassays to in-country laboratories at the Medical Research Council Unit The Gambia (MRCG), allowing the majority of data generation and analysis to be performed locally. Training and mentorship for local researchers were integral to the study, and two Gambian scientists, F.C. and O.C., were subsequently awarded PhD fellowships. A.J.K., E.P.A. and O.C. are members, or alumni, of the Wellcome CREATE PhD program, an equity-centred doctoral training initiative focused on fostering partner-led, inclusive global health research practices.

Equitable benefit sharing was considered at every stage of the research. There was no transfer of biological materials out of The Gambia without prior ethics committee approval and participant informed consent, in alignment with national governance and international principles, such as the Nagoya Protocol. To support ethical engagement with participants, preliminary findings from the study were shared with community members and local leaders through a culturally appropriate, locally delivered community engagement event.

### Reporting summary

Further information on research design is available in the Nature Portfolio Reporting Summary linked to this article.

## Online content

Any methods, additional references, Nature Portfolio reporting summaries, source data, extended data, supplementary information, acknowledgements, peer review information; details of author contributions and competing interests; and statements of data and code availability are available at 10.1038/s41591-025-03868-4.

## Supplementary information

Supplementary Information Supplementary Figs. 1–7, Tables 1 and 2, and Note.

Reporting Summary

## Figures and Tables

**Extended Data Table 1:**
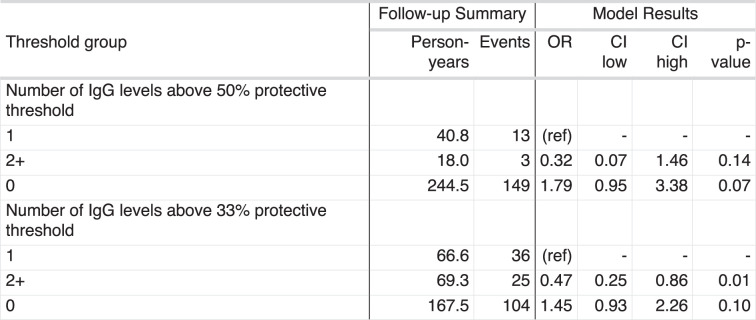
Follow-up time and protective association of IgG levels above combined protective thresholds

^1^CI, confidence interval; HR, hazard ratio.

IgG levels were measured from *n* = 1,987 visits. At each timepoint, the number of IgG levels above 50% and 33% putative protective thresholds were calculated (range, 0–3). Logistic regression analysis was performed with mixed effects to explore the relationship between the number of IgG levels above protective thresholds and culture-confirmed events within 45 days.

**Extended Data Table 2:**
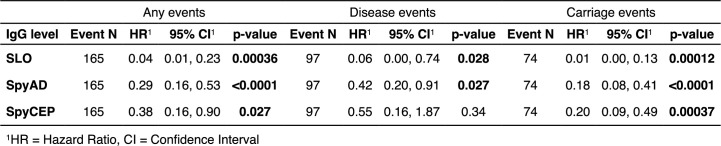
HRs of incidence of *S. pyogenes* events by blood IgG level above the transition point, adjusted for sex, age group and household size

^1^CI, confidence interval; HR, hazard ratio. IgG measurements from 1,987 timepoints were included in multivariable Cox proportional hazards models to explore the impact of IgG levels on incident culture-confirmed carriage and disease. Antibody levels above the transition point of 4.3 log_10_ RLU ml^−1^ for SLO, 4.1 log_10_ RLU ml^−1^ for SpyAD and 4.3 log_10_ RLU ml^−1^ for SpyCEP were included alongside age group, sex and household.

**Extended Data Table 3:**
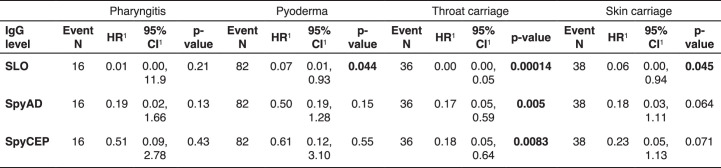
HRs of incidence of *S. pyogenes* events by blood IgG level above the transition point, adjusted for sex, age group and household size, including a detailed event breakdown

^1^CI, confidence interval; HR, hazard ratio. IgG measurements from 1,987 timepoints were included in multivariable Cox proportional hazards models to explore the impact of IgG levels on incident culture-confirmed events by event type. Antibody levels above the transition point of 4.3 log_10_ RLU ml^−1^ for SLO, 4.1 log_10_ RLU ml^−1^ for SpyAD and 4.3 log_10_ RLU ml^−1^ for SpyCEP were included alongside age group, sex and household.

**Extended Data Table 4:**
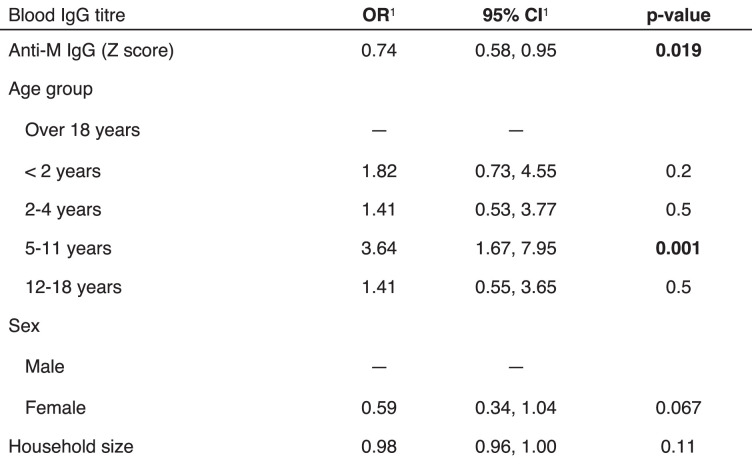
Cluster-related blood IgG and probability of any event in the next 45 days

^1^CI, confidence interval; OR, odds ratio. Anti-M IgG before, during and after culture-confirmed, M/*emm* type characterized events in both cases (*n* = 378 measurements, 143 events, 103 participants) and household controls (*n* = 1,366 measurements from 293 participants) was measured against the cluster-related M peptide to the M/*emm* type of the event. The cluster-related measurement was the direct homologous M peptide wherever available; otherwise, the cluster-homologous M peptide was used. The association between *z*-score normalized cluster-related anti-M IgG and the occurrence of any event within the next 45 days was explored using logistic mixed-effects models, incorporating age group, sex and household size.

## Data Availability

Anonymized data as an open resource for the research community to reproduce and extend analyses are publicly available at Zenodo: 10.5281/zenodo.14887949. Data are published under a Creative Commons Attribution 4.0 International license. Requests for additional study metadata (for example, detailed individual participant metadata and social mixing data) can be made and will be considered upon formal request to the corresponding authors.

## Code availability

The code to reproduce analyses from publicly available data is available at https://github.com/Clinical-Infection-Research-UoSheffield/Development_natural_protective_immunity_Streptococcus_pyogenes.
