# Publisher Correction: Early-life serological profiles and the development of natural protective humoral immunity to *Streptococcus pyogenes* in a high-burden setting

**DOI:** 10.1038/s41591-025-04017-7

**Published:** 2025-09-24

**Authors:** Alexander J. Keeley, Fatoumata E. Camara, Edwin P. Armitage, Gabrielle de Crombrugghe, Jainaba Sillah, Modou Lamin Fofana, Victoria Rollinson, Elina Senghore, Musukoi Jammeh, Alana L. Whitcombe, Amat Bittaye, Haddy Ceesay, Isatou Ceesay, Bunja Samateh, Muhammed Manneh, Martina Carducci, Luca Rovetini, Elena Boero, Luisa Massai, Lady Chilel Sanyang, Ousman Camara, Ebrima E. Cessay, Miren Iturriza, Danilo Gomes Moriel, Adam Kucharski, Pierre R. Smeesters, Anne Botteaux, Ya Jankey Jagne, Nicole J. Moreland, Ed Clarke, Beate Kampmann, Michael Marks, Omar Rossi, Henrik Salje, Claire E. Turner, Thushan I. de Silva

**Affiliations:** 1 Vaccines and Immunity Theme, MRC Unit The Gambia at the London School of Hygiene and Tropical Medicine, Fajara, The Gambia; 2 Division of Clinical Medicine, School of Medicine and Population Health, University of Sheffield, Sheffield, UK; 3 The Florey Institute of Infection, University of Sheffield, Sheffield, UK; 4 Department of Clinical Research, London School of Hygiene and Tropical Medicine, London, UK; 5 Population Health Sciences, Bristol Medical School, University of Bristol, Bristol, UK; 6 Molecular Bacteriology Laboratory, European Plotkin Institute for Vaccinology, Université Libre de Bruxelles, Brussels, Belgium; 7 Department of Paediatrics, Brussels University Hospital, Academic Children Hospital Queen Fabiola, Université Libre de Bruxelles, Brussels, Belgium; 8 Faculty of Medical and Health Sciences, The University of Auckland, Auckland, New Zealand; 9 GSK Vaccines Institute for Global Health (GVGH), Siena, Italy; 10 Centre for Mathematical Modelling of Infectious Diseases, London School of Hygiene and Tropical Medicine, London, UK; 11 Charité Centre for Global Health, Charité – Universitätsmedizin Berlin, Berlin, Germany; 12 Hospital for Tropical Diseases, University College London Hospital, London, UK; 13 Division of Infection and Immunity, University College London, London, UK; 14 Department of Genetics, University of Cambridge, Cambridge, UK; 15 School of Biosciences, University of Sheffield, Sheffield, UK

**Keywords:** Bacterial infection, Bacteriology, Translational immunology

Correction to: *Nature Medicine* 10.1038/s41591-025-03868-4, published online 8 August 2025.

In the version of the article initially published, in the “Early-life serological profiles” section, the text “…two or more antigens of greater than 0.5 log10 RLU ml^−1^” should have read “…two or more antigens or greater than 0.5 log10 RLU ml^−1^”. Additionally, in the “M/*emm* type-specific IgG measurement in a multiplex assay” section of the Methods, the sentence “Microspheres were conjugated to streptavidin-coupled MagPlex beads…” has been corrected to “Biotinylated peptides were conjugated to streptavidin-coupled MagPlex beads…”. These corrections have been made to the HTML and PDF versions of the article.

