# Supplementary material for: Early-life serological profiles and the development of natural protective humoral immunity to Streptococcus pyogenes in a high-burden setting
Source: Nat Med. 2025 Aug 8;31(10):3360–71. doi: 10.1038/s41591-025-03868-4 (PMC12532705; doi:10.1038/s41591-025-03868-4)
Supplement: Supplementary file 1 — Supplementary Figs. 1–7, Tables 1 and 2, and Note. [file 41591_2025_3868_MOESM1_ESM.pdf]

nature medicine

Supplementary information https://doi.org/10.1038/s41591-025-03868-4

Early-life serological profiles and the development of natural protective humoral immunity to *Streptococcus pyogenes* in a high-burden setting

In the format provided by the authors and unedited

Supplementary material: Early life serological profiles and the development of natural protective humoral immunity to *Streptococcus pyogenes* in a high burden setting

Figures:

Supplementary Figure 1: Breakdown of culture confirmed S. pyogenes events in the SpyCATS study. 2
Supplementary Figure 2: Comparison of fetal:maternal IgG transfer ratio between infants with and without serological evidence of exposure between 6months and subsequent visit. ... 3
Supplementary Figure 3: Correlation of IgG levels to conserved antigens within individuals . 4
Supplementary Figure 4: Correlation between IgG levels within individuals at different age strata 6
Supplementary Figure 5: Placental transfer of IgG to emm cluster-representative M peptides. 7
Supplementary Figure 6: Correlation between IgG level measurements in serum and dried blood spot 8
Supplementary Figure 7: THP-1 gating for a representative positive sample and negative sample. 11

Tables:

Supplementary Table 1: Breakdown of events with pre and post event IgG levels measured by age group....... 5
Supplementary Table 2: M peptides included in M/emm-type specific IgG assays 9

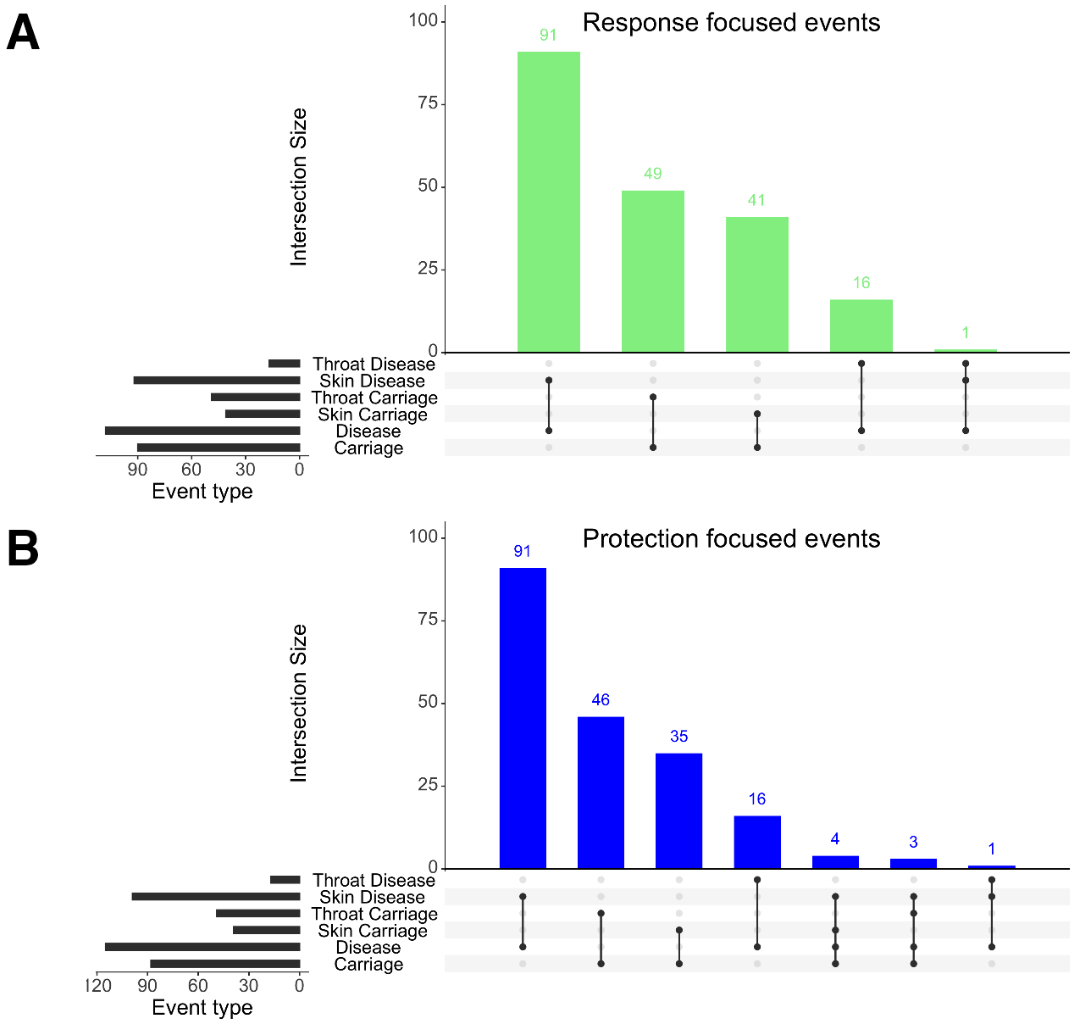

Supplementary Figure 1: Breakdown of culture confirmed S. pyogenes events in the SpyCATS study.

(A): Breakdown of response focuses events (RFEs) in the SpyCATS study. 198 culture confirmed *S. pyogenes* events were defined as RFEs. RFEs were defined to study immune responses to specific events. Disease events were defined as presence of signs or symptoms of pharyngitis or pyoderma plus a positive culture for *S. pyogenes* from the disease site. Carriage events were defined as detection of *S. pyogenes* from throat or skin swabs without symptoms or signs of disease. RFE carriage events could only be defined in absence of a disease event within preceding 42 or following 14 days. (B) Breakdown of protection focused events (PFEs) in the SpyCATS study. 196 culture confirmed *S. pyogenes* events were defined as PFEs. PFEs were defined previously for the purpose of risk factor analysis. We used PFEs to analyze immune protection against incident events. Disease PFEs defined as presence of signs or symptoms of pharyngitis or pyoderma plus a positive culture for *S. pyogenes* from the disease site. Carriage was defined as a positive culture for *S. pyogenes* from a site with no disease, but could occur simultaneously to disease at another site (in contrast to RFEs). *S. pyogenes* positive culture at study baseline was not considered a PFE for incident event analysis. The intersection size represents the number of events shared between the types of event.

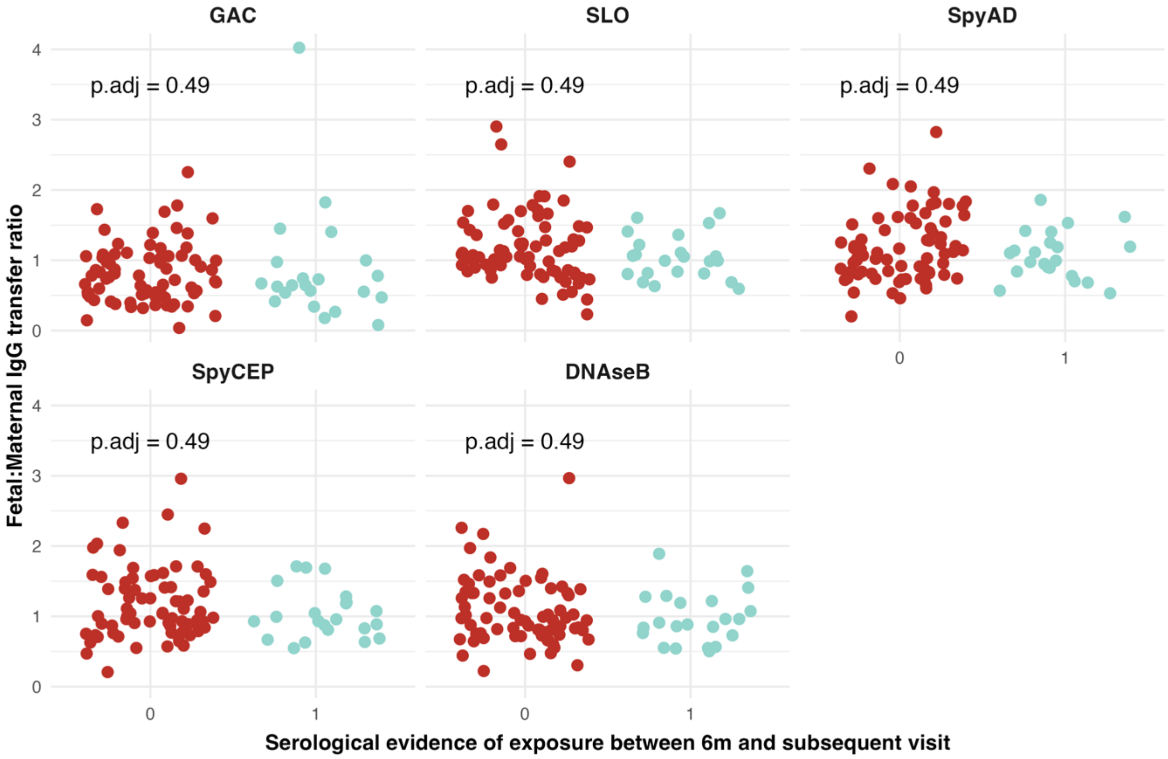

Supplementary Figure 2: Comparison of fetal:maternal IgG transfer ratio between infants with and without serological evidence of exposure between 6months and subsequent visit.

Paired maternal and cord blood IgG titres were measured in n=94 mother child pairs at delivery. Fetal maternal transfer ratio (F:MR) was calculated, with levels <1 signifying incomplete transfer of IgG to the newborn. (F:MR) values between the two groups were compared using a paired Wilcoxon signed-rank test. P-values were adjusted for multiple testing using the false discovery rate (FDR) correction.

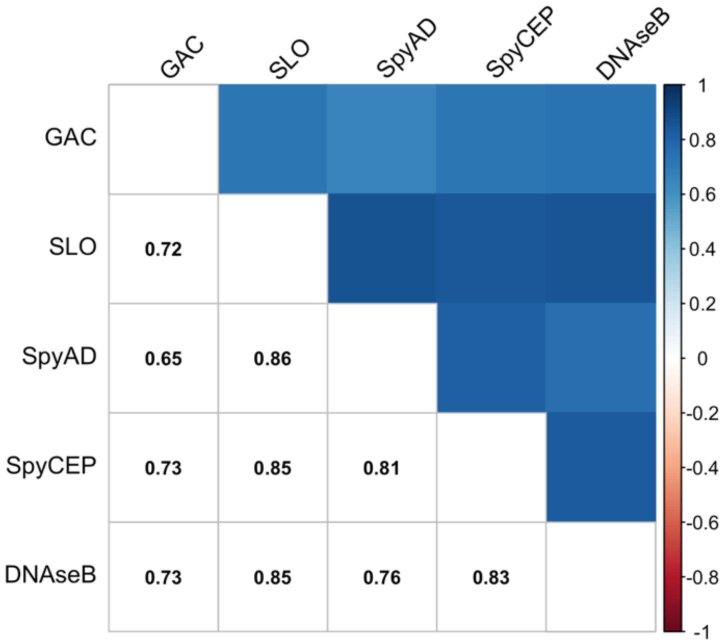

Supplementary Figure 3: Correlation of IgG levels to conserved antigens within individuals

Pairwise correlation coefficients (Pearson's method) comparing IgG levels in blood and for individual participants from baseline samples, in those with no disease events at baseline visit, in the SpyCATS longitudinal cohort study (n=413, P < 0.0001 for all comparisons).

Supplementary Table 1: Breakdown of events with pre and post event IgG levels measured by age group.

**Characteristic**	**Overall** N = 150[1]	**< 2 years** N = 28[1]	**2-4 years** N = 22[1]	**5-11 years** N = 66[1]	**12-18 years** N = 19[1]	**Over 18 years** N = 15[1]
event_type						
other	1	0 (0%)	0 (0%)	1 (100%)	0 (0%)	0 (0%)
skin carriage	35	6 (17%)	6 (17%)	17 (49%)	4 (11%)	2 (5.7%)
skin disease	67	18 (27%)	9 (13%)	28 (42%)	8 (12%)	4 (6.0%)
throat carriage	34	4 (12%)	6 (18%)	13 (38%)	5 (15%)	6 (18%)
throat disease	13	0 (0%)	1 (7.7%)	7 (54%)	2 (15%)	3 (23%)

[1]n (%)

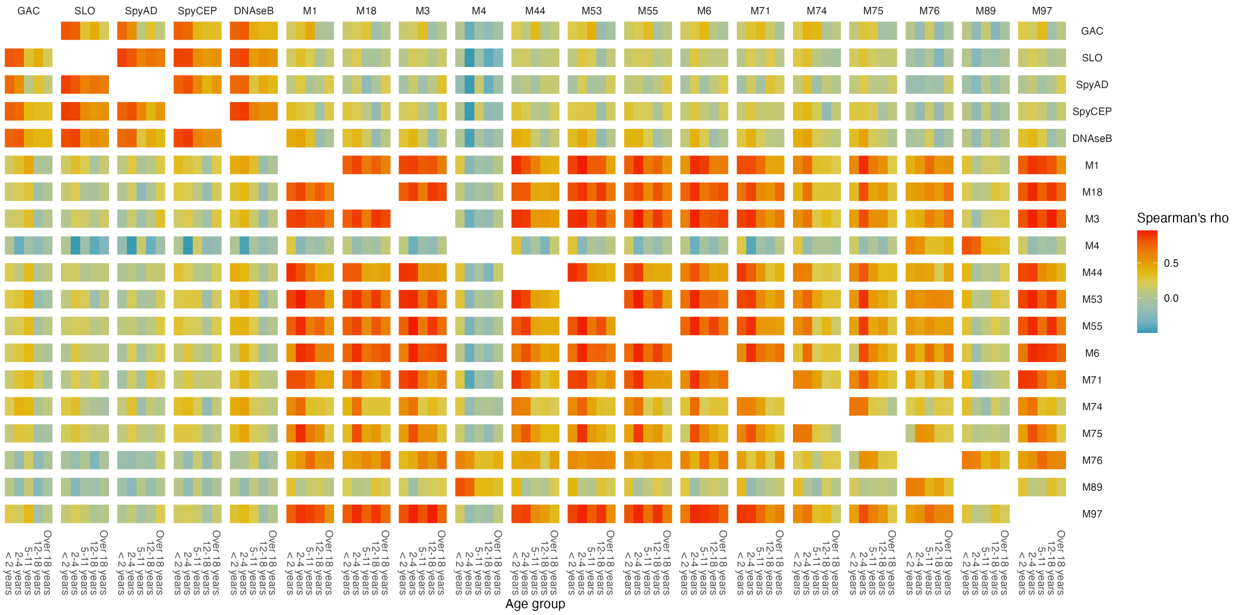

Supplementary Figure 4: Correlation between IgG levels within individuals at different age strata

Correlation between antigen-specific IgG profiles in those with no disease events at baseline visit, in sera from the SpyCATS cohort study (n=402) by age group. The correlation between IgG within individuals in each age group was determined with Spearman's method.

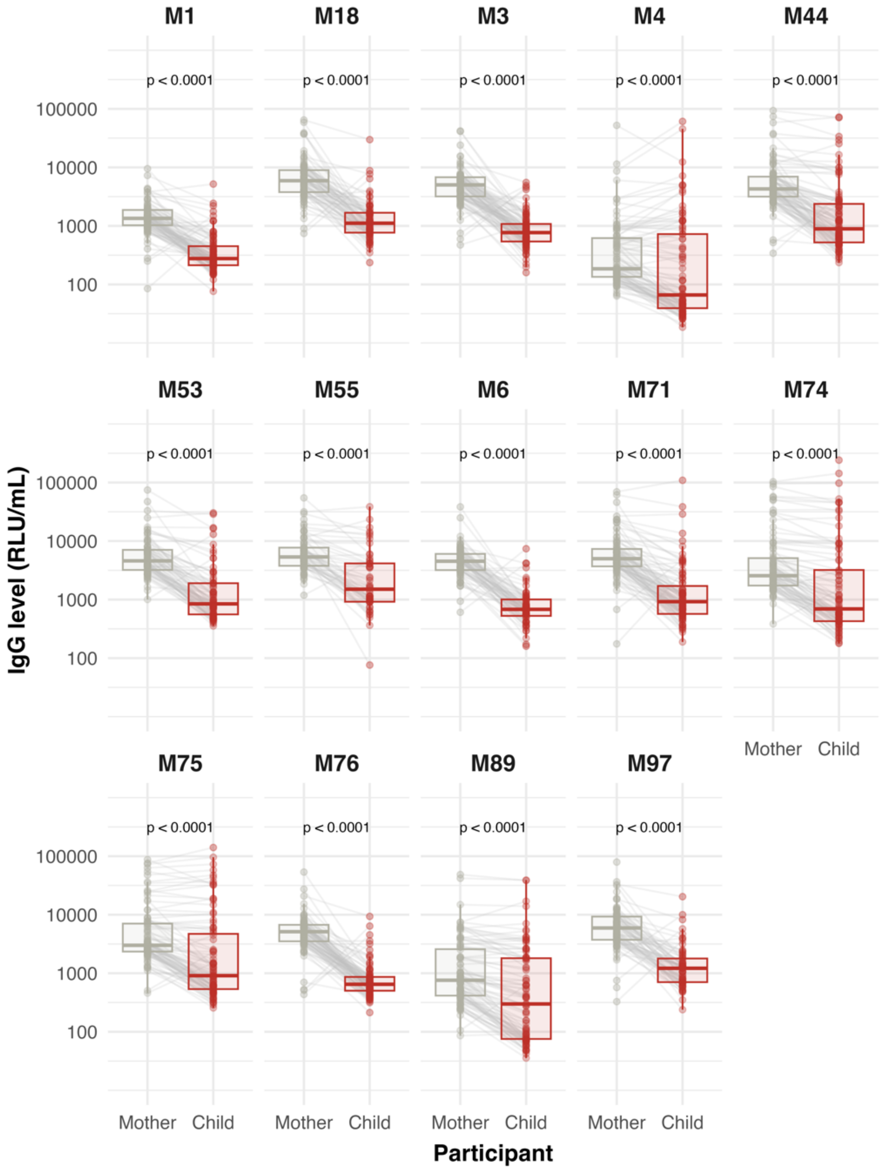

Supplementary Figure 5: Placental transfer of IgG to emm cluster-representative M peptides.

IgG levels in n=94 paired maternal serum and neonatal cord blood at delivery. IgG levels were compared using a paired Wilcoxon signed-rank test. P-values were adjusted for multiple testing using the false discovery rate (FDR) correction.

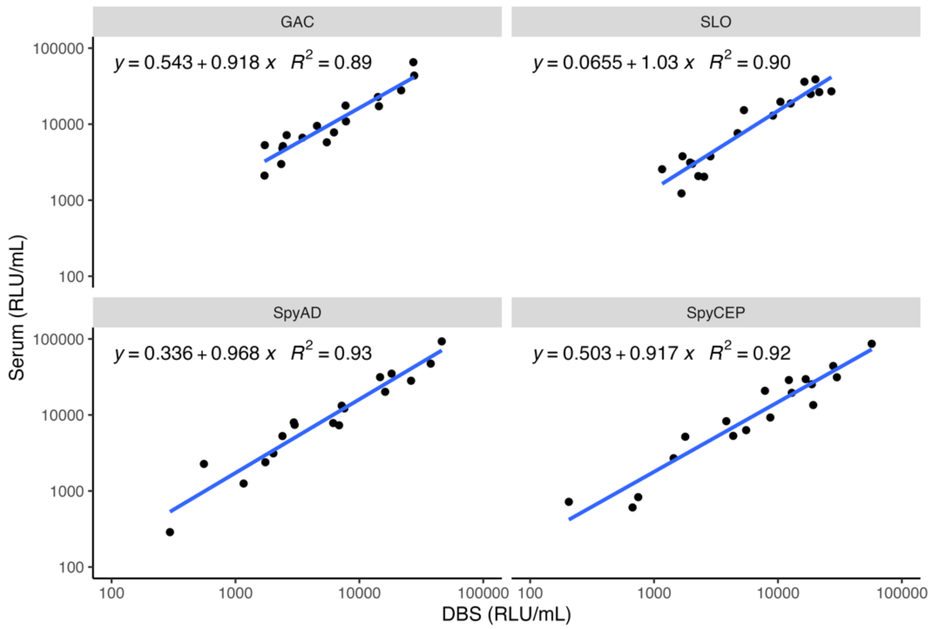

Supplementary Figure 6: Correlation between IgG level measurements in serum and dried blood spot

IgG levels from adult healthcare workers (n=18) with paired serum and eluted dried blood spot (DBS) samples were measured. Log transformed IgG levels (RLU/mL) measured by Luminex 4-plex assay were compared with Spearman's method. Correlation coefficient and regression equation are given for each antigen. Blue line represents the line of best fit. $P < 0.0001$ for all comparisons.

Supplementary Table 2: M peptides included in M/emm-type specific IgG assays

Emm type	*Emm* Cluster	In 30 valent vaccine	Bead region	Cluster representative * assay	E3 cluster assay
emm1	A-C3	Y	19	Y	N
emm12	A-C4	Y	NA *	NA*	N
emm18	Singleton	Y	51	Y	N
emm3	A-C5	Y	54	Y	N
emm4	E1	Y	75	Y	N
emm44	E3	Y	57	Y	Y
emm53	D4	N	45	Y	N
emm55	Singleton	N	26	Y	N
emm6	Singleton	Y	21	Y	N
emm71	D2	N	36	Y	N
emm74	Singleton	Y	72	Y	N
emm75	E6	Y	27	Y	N
emm76	E2	Y	30	Y	N
emm89	E4	Y	78	Y	N
emm97	D5	N	38	Y	N
emm103	E3	N	33	N	Y
emm113	E3	N	67	N	Y
emm25	E3	N	48	N	Y
emm82	E3	Y	61	N	Y
emm87	E3	Y	42	N	Y

* The 15 most common M/*emm* clusters, based on worldwide population data, were determined. For each M/*emm* cluster, the most frequent *emm* type globally was selected as the cluster-representative M peptide. M12 identified as cluster representative M peptide for M/*emm* cluster A-C4 but peptide not available for inclusion in multiplex assay leaving 14 HVRs in the cluster representative multiplex assay.

Supplementary Note: M Peptide sequences

```
Cluster representative M peptides:

>89
DSDNINRSVSVKDNEKELHNKIADLEEERGEHLDKIDELKEELKAKEKSS
>44
AESRSVSQGSVSLELYDKLSDENDILREKQDEYLTKIDGLDKENKEYASQ
>1
NGDGNPREVIEDLAANNPAIQNIRLRHENKDLKARLENAMEVAGRDFKRA
>75
EEERTFTELPYEARYKAWKSENDELRENYRRTLDKFNTEQGKTTRLEEQN
>12
DHSDLVAEKQRLEDLGQKFERLKQRSELYLQQYYDNKSNGYKGDWYVQQL
>53
NRADDARNEVLRGNLVRAELWYRQIQENDQLKLENKGLKTDLREKEEELQ
>76
ADANSKSVSNSNVSINLYNELQAEHDKLQTKHEELLAEHDALKEKQDKNQ
>4
AEIKKPQADSAWNWPKEYNALLKENEELKVEREKYLSYADDKEKDPQYRA
>3
DARSVNGEFPRHVKLKNEIENLLDQVTQLYTKHNSNYQQYNAQAGRLDLR
>6
RVFPRGTVENPDKARELLNKYDVENSMLQANNDKLTTENKNLTDQNKNLT
>71
RAITRATSDDPAKLKQMVEGYELENHTLKNDKEKLTTENSALTTEKNRLT
>74
FTVTRSMTRDYLAKVVQDFDTKNHELETHNSELSATNQTLQGQVEAEQKK
>18
APLTRATADNKDELIKRANDYEIQNHQLTVENKKLKTDKEQLTKENDDLK
>55
NQTEPSQTNNRLYQERQRLQDLKSKFQDLKNRSEGYIQQYYDEEKNSGSN
>97
DNGRAIYERARERALQELGPVPRRLWLREYEKNQELTKKLTEFEEKLLQN

Additional E3 peptides:

>103
DSPRDVTSDLTTSMWKKKAEEAEAKASKFEKQLEDYKKAQKDYYEIEEKL
>113
ENRQSVSNGGSVSIDQYNKLSDERNNLLDQNGDLLDQNEILRKKQEELEK
>25
DEGPKDITDSLPAPMWRDKAKAAEAKVDKLEKQLEGYKKLEEDYFNLEKR
>82
DSSSRDITEAGVSKFWKSKFDAEQNRANELEKKLSGYEKDYKTLEQEYEN
>87
ESPREVTNELAASVWKKKVEEAKEKASKLEKQLEEAQKDYSEIEGKLEQF
```

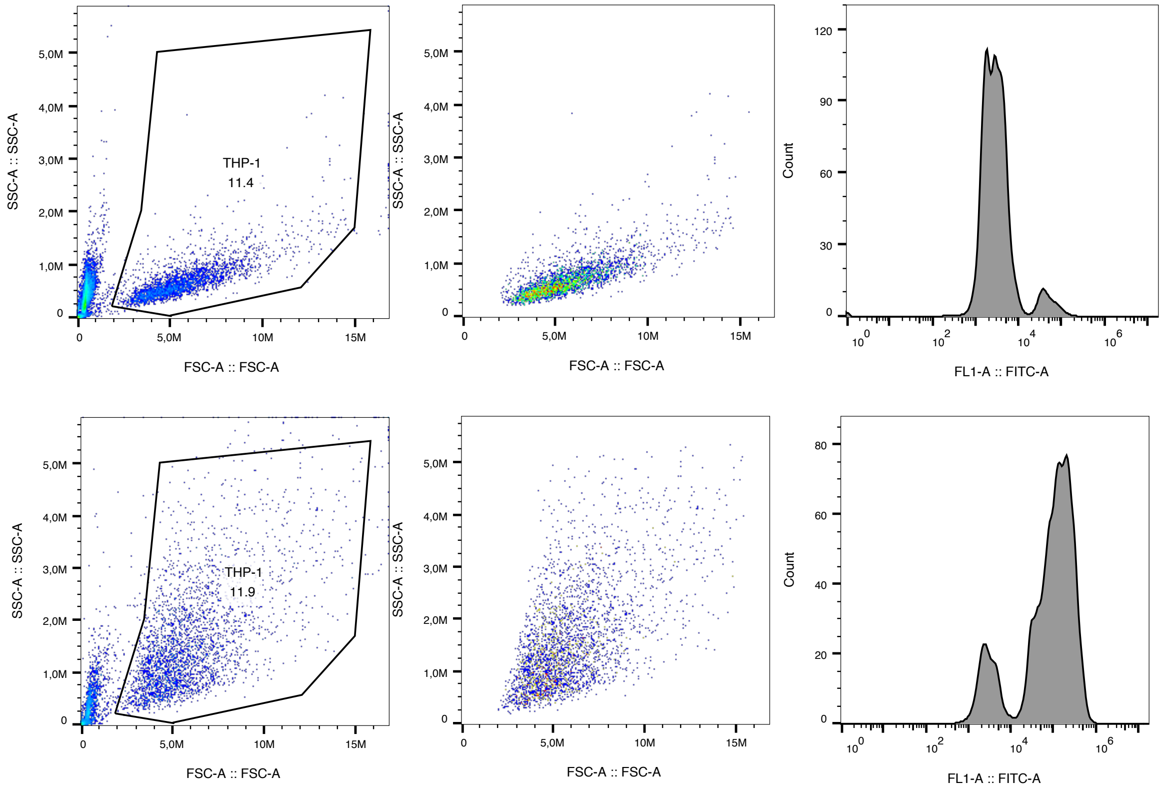

Supplementary Figure 7: THP-1 gating for a representative positive sample and negative sample.

Top panel consists of representative flow cytometric output from an experiment with M1 bacteria, THP-1 cells and sample serum. Bottom panel consists of representative flow cytometric output from an experiment with M1 bacteria, THP-1 cells and IgG-depleted serum.
