# Supplementary material for: Early-life serological profiles and the development of natural protective humoral immunity to Streptococcus pyogenes in a high-burden setting
Source: Nat Med. 2025 Aug 8;31(10):3360–71. doi: 10.1038/s41591-025-03868-4 (PMC12532705; doi:10.1038/s41591-025-03868-4)
Supplement: Supplementary file 2 — Reporting Summary [file 41591_2025_3868_MOESM2_ESM.pdf]

nature portfolio

Corresponding author(s): Alexander Keeley, Thushan de Silva

Last updated by author(s): May 15, 2025

# Reporting Summary

Nature Portfolio wishes to improve the reproducibility of the work that we publish. This form provides structure for consistency and transparency in reporting. For further information on Nature Portfolio policies, see our Editorial Policies and the Editorial Policy Checklist.

## Statistics

For all statistical analyses, confirm that the following items are present in the figure legend, table legend, main text, or Methods section.

| n/a | Confirmed | |
|---|---|---|
| ☐ | ☒ | The exact sample size (*n*) for each experimental group/condition, given as a discrete number and unit of measurement |
| ☐ | ☒ | A statement on whether measurements were taken from distinct samples or whether the same sample was measured repeatedly |
| ☐ | ☒ | The statistical test(s) used AND whether they are one- or two-sided<br>*Only common tests should be described solely by name; describe more complex techniques in the Methods section.* |
| ☐ | ☒ | A description of all covariates tested |
| ☐ | ☒ | A description of any assumptions or corrections, such as tests of normality and adjustment for multiple comparisons |
| ☐ | ☒ | A full description of the statistical parameters including central tendency (e.g. means) or other basic estimates (e.g. regression coefficient) AND variation (e.g. standard deviation) or associated estimates of uncertainty (e.g. confidence intervals) |
| ☐ | ☒ | For null hypothesis testing, the test statistic (e.g. *F*, *t*, *r*) with confidence intervals, effect sizes, degrees of freedom and *P* value noted<br>*Give P values as exact values whenever suitable.* |
| ☒ | ☐ | For Bayesian analysis, information on the choice of priors and Markov chain Monte Carlo settings |
| ☒ | ☐ | For hierarchical and complex designs, identification of the appropriate level for tests and full reporting of outcomes |
| ☐ | ☒ | Estimates of effect sizes (e.g. Cohen's *d*, Pearson's *r*), indicating how they were calculated |

*Our web collection on statistics for biologists contains articles on many of the points above.*

## Software and code

Policy information about availability of computer code

| | |
|---|---|
| Data collection | Bio-Plex Manager version 6.2 was used for Luminex data collection and interpolation of median fluorescent intensity (MFI) from standard curves. |
| Data analysis | R version 4.4.0 for all analysis except IC50 data during functional assays which were produced in Graphpad (v10), and in flow cytometry which were analysed in FlowJo (Beckton Dickinson & Company, version 10.9.0). |

For manuscripts utilizing custom algorithms or software that are central to the research but not yet described in published literature, software must be made available to editors and reviewers. We strongly encourage code deposition in a community repository (e.g. GitHub). See the Nature Portfolio guidelines for submitting code & software for further information.

## Data

Policy information about availability of data

All manuscripts must include a data availability statement. This statement should provide the following information, where applicable:

- Accession codes, unique identifiers, or web links for publicly available datasets
- A description of any restrictions on data availability
- For clinical datasets or third party data, please ensure that the statement adheres to our policy

Anonymised data as an open resource for the research community to reproduce and extended analyses is publicly available at Zenodo: https://doi.org/10.5281/zenodo.14887949. Data are published under a Creative Commons Attribution 4.0 International license. Requests for additional study metadata (eg detailed individual participant metadata, social mixing data) can be made and will be considered on formal request to corresponding authors.

## Research involving human participants, their data, or biological material

Policy information about studies with human participants or human data. See also policy information about sex, gender (identity/presentation), and sexual orientation and race, ethnicity and racism.

**Reporting on sex and gender**

We report sex as a demographic characteristic of our cohort.
In the household cohort study: 233 (58%) of participants were female participants.
In mother child cohort all mothers were female and with 35 (37%) of children were female.
Our principle analyses (exploring the association of IgG antibodies with protection) include sex as a covariate.
Sex was self reported by participants at enrollment.

**Reporting on race, ethnicity, or other socially relevant groupings**

In this manuscript no reference is made to race, ethnicity, or other socially relevant grouping.

**Population characteristics**

Age, age group, household size, sex variables were collected and used as covariates in our analyses.

Age range was 0-85. The median age was 15 years (IQR 6–28), 233 (53%) were female, and the median household size was 7 (IQR 6–10).

**Recruitment**

We performed a prospective, longitudinal, household cohort study in the urban area of Sukuta, The Gambia, over a 13-month period in 2021–22. Households containing at least three members, including one child younger than 18 years, were eligible for inclusion. Households were excluded if more than 50% of household members declined to participate. All individuals residing in the households were invited to participate, with the exclusion of those with any condition or circumstance that might cause difficulty or discomfort in sample collection, or those deemed by a study team member as unable or unlikely to adhere to the study protocol. Households were identified by random GPS selection (appendix p 14). Random GPS coordinates within the boundaries of Sukuta were derived from 2013 census data using QGIS version 3.12, stratified by low, medium, and high housing density areas. For each set of GPS coordinates, the nearest household was approached for participation, until the target number of households was met.

These rigorous steps in recruitment sought to minimise sampling bias.

Reference DOI: 10.1016/S2666-5247(24)00046-6:

**Ethics oversight**

The studies received approval from the joint ethics committee of The Gambia Government/Medical Research Council and the London School of Hygiene & Tropical Medicine Research Ethics Committee (ref: 24005 and 1585). Written informed consent was obtained from adult participants, as well as from parents or guardians for participants under 18 years of age. Additionally, children aged 12 to 17 years provided assent. The studies are registered on ClinicalTrials.gov (NCT05117528 and NCT03746665).

Note that full information on the approval of the study protocol must also be provided in the manuscript.

# Field-specific reporting

Please select the one below that is the best fit for your research. If you are not sure, read the appropriate sections before making your selection.

☒ Life sciences ☐ Behavioural & social sciences ☐ Ecological, evolutionary & environmental sciences

For a reference copy of the document with all sections, see nature.com/documents/nr-reporting-summary-flat.pdf

# Life sciences study design

All studies must disclose on these points even when the disclosure is negative.

**Sample size**

Sample size considerations
The primary outcome measures used to determine sample size were:

1.Monthly S. pyogenes carriage prevalence, and
2.S. pyogenes carriage and infection incidence over 12 months.

In HIC, S pyogenes pharyngeal carriage prevalence in children is 2–17% , and in Uganda is 15.9% . Our study also includes adults, in whom carriage is lower, but will use pooled skin and pharyngeal carriage as our outcome measure, which will likely increase prevalence in turn. We therefore estimate a pooled prevalence of 15%.

S. pyogenes pharyngeal carriage yearly incidence in children in the US was shown to be 27–32%. We found a skin infection incidence of 592/1000 child years in The Gambia during an influenza vaccine study follow-up (unpublished data) and of which ~50% are likely due to S. pyogenes.. As we are including adults with a likely lower incidence, we estimate a yearly incidence of 20%.

The sample size was calculated for the primary objective, S. pyogenes carriage prevalence, using the formula below to measure the estimated prevalence of 15% with a precision of ±5%.

Using Z=1.96 for α=0.05, p=0.15 and e=0.05 we require a sample of 196. Intracluster correlation is unknown, therefore we used a conservative design effect of 2, which allowing for 10% drop-out rate gives a required sample size of 431.

We therefore propose to recruit 45 households, which with an average household size of 10, will equal approximately 450 individuals for the main cohort.

This sample size would provide adequate power for precise estimates of prevalence and incidence of StrepA carriage (precision between ±4 and ±5%) and to detect risk factors for S. pyogenes carriage with prevalence (or incidence) rate ratios of greater than 2 with 80% power.

See reference DOI: https://doi.org/10.12688/wellcomeopenres.18716.2

**Data exclusions**

Sample availability determined the number of datapoints in each analysis in the manuscript and are described in respective figure legend and in results sections. No systematic data exclusion

**Replication**

Our principle analysis exploring the association between IgG levels and culture confirmed S. pyogenes events were performed using two orthagonal approaches with sensitivity analyses reported in manuscript and supplementary information section.

Replication of findings via orthagonal approaches confirmed the main findings.

Reproducibility of assays used in this manuscript have been characterised in four unique methods papers, referenced throughout the manuscript.

Keeley, A. J. et al. Development and Characterisation of a Four-Plex Assay to Measure Streptococcus pyogenes Antigen-Specific IgG in Human Sera. Methods Protoc. 5, 55 (2022).

Carducci, M. et al. Development and characterization of a hemolysis inhibition assay to determine functionality of anti-Streptolysin O antibodies in human sera. J. Immunol. Methods 526, 113618 (2024).

Massai, L. et al. Characterization of an IL-8 cleavage inhibition assay to determine the functionality of anti-SpyCEP antibodies in human sera. J. Immunol. Methods 536, 113786 (2025).

Boero, E. et al. A flow cytometry-based assay to determine the ability of anti-Streptococcus pyogenes antibodies to mediate monocytic phagocytosis in human sera. J. Immunol. Methods 528, 113652 (2024).

**Randomization**

The randomisation proceudre for recruitment into the study is provided: DOI: 10.1016/S2666-5247(24)00046-6:

Households were identified by random GPS selection (appendix p 14). Random GPS coordinates within the boundaries of Sukuta were derived from 2013 census data using QGIS version 3.12, stratified by low, medium, and high housing density areas. For each set of GPS coordinates, the nearest household was approached for participation, until the target number of households was met.

**Blinding**

No blinding was performed given that this was a longitudinal cohort study. Laboratory team members were blinded to clinical study outcomes when delivering immunological assays.

# Reporting for specific materials, systems and methods

We require information from authors about some types of materials, experimental systems and methods used in many studies. Here, indicate whether each material, system or method listed is relevant to your study. If you are not sure if a list item applies to your research, read the appropriate section before selecting a response.

## Materials & experimental systems

| n/a | Involved in the study |
|---|---|
| | ☒ Antibodies |
| | ☒ Eukaryotic cell lines |
| ☒ | ☐ Palaeontology and archaeology |
| ☒ | ☐ Animals and other organisms |
| | ☒ Clinical data |
| ☒ | ☐ Dual use research of concern |
| ☒ | ☐ Plants |

## Methods

| n/a | Involved in the study |
|---|---|
| ☒ | ☐ ChIP-seq |
| | ☒ Flow cytometry |
| ☒ | ☐ MRI-based neuroimaging |

# Antibodies

Antibodies used

R-Phycoerythrin AffiniPure F(ab')$_2$ Fragment Goat Anti-Human IgG, F(ab')$_2$ fragment specific. Jackson ImmunoResearch.(109-116-097)
Antibodies (for IL8 assay) - human IL - 8 Immunoassay ELIS A kit (Invitrogen -Cat #KAC1301)

Validation

R-Phycoerythrin AffiniPure F(ab')$_2$ Fragment Goat Anti-Human IgG, F(ab')$_2$ fragment specific. Jackson ImmunoResearch.
(109-116-097): Antibody Specificity: Based on immunoelectrophoresis and/or ELISA, the antibody reacts with the F(ab')2/Fab portion of human IgG.
It also reacts with the light chains of other human immunoglobulins. No antibody was detected against the Fc portion of human IgG or against non-immunoglobulin serum proteins. The antibody has been tested by ELISA and/or solid-phase adsorbed to ensure minimal cross-reaction with bovine, horse, and mouse serum proteins, but it may cross-react with immunoglobulins from other species.

Obtained from: https://www.jacksonimmuno.com/catalog/products/109-116-097

Antibodies (for IL8 assay) - human IL - 8 Immunoassay ELIS A kit (Invitrogen -Cat #KAC1301):Analytical information: This product has been tested by Quality Control and passed internal specifications.

Obtained from: https://www.thermofisher.com/elisa/product/Mouse-IL-18-Uncoated-ELISA-Kit-with-Plates/88-50618-22

# Eukaryotic cell lines

Policy information about cell lines and Sex and Gender in Research

Cell line source(s)

Myeloid leukaemia cell line THP-1 was purchased from ATCC (ATCC TIB-202) LOT:70053863

Authentication

Obtained from ATCC: https://www.atcc.org/products/tib-202:

Authentication performed by ATCC:

Name:THP-1
Description:Acute Monocytic Leukemia
Species:Human (Homo sapiens)
Volume/Ampule: Approximately 1 mL
Date Frozen: 07JUL2020
Recovery:A T-75 setup at a seeding density of 4.0 x 105 viable cells/mL reaches approximately 7.9 x 105 viable cells/mL in 3 days and 1.0 x 106 viable cells/mL in 6 days with media addition.

Mycoplasma contamination

Obtained from ATCC: https://www.atcc.org/products/tib-202:

Test for mycoplasma contamination
Hoechst DNA stain (indirect) method: None detected
Agar culture (direct) method: None detected
PCR-based assay: None detected

Commonly misidentified lines
(See ICLAC register)

No commonly misidentified cell lines were used in the study

# Clinical data

Policy information about clinical studies

All manuscripts should comply with the ICMJE guidelines for publication of clinical research and a completed CONSORT checklist must be included with all submissions.

Clinical trial registration

The studies are registered on ClinicalTrials.gov (NCT05117528 and NCT03746665).

Study protocol

Study protocol:
https://wellcomeopenresearch.org/articles/8-41/v2

Data collection

Data collected from a prospective, longitudinal, household cohort study in the urban area of Sukuta, The Gambia, over a 13-month period in 2021–22.

We recruited 337 participants from 44 households between July 27 and Sept 2, 2021, at MV0. An additional 105 participants from the same households were recruited at subsequent monthly visits, resulting in a total of 442 participants. Final visits were conducted between June 28 and Sept 28, 2022.

Outcomes

Primary outcomes of the study:

1. To determine the prevalence, incidence, duration and transmission dynamics of asymptomatic StrepA carriage and clinical StrepA infections within households.

Measured by clinical assessment, microbiological culture for Group A Streptococci from throat, skin and pyoderma lesions.

2. To establish risk factors for pharyngeal and skin clinical StrepA infection, including detailed characterisation of the relationship with individual and household asymptomatic carriage, emm type and seasonality.

Risk factors measured by study team delivered questionaires. Assessed with Cox Proportional Hazards Models

Relevant secondary objetives:

1. To describe age-stratified anti-StrepA antibody titres.

Assessed by measurement of antibodies to streptococcal antigens

2. To explore StrepA-specific serological and mucosal immune activity in response to colonisation and disease.

Assessed by measurement of antibodies to streptococcal antigens combined with clinical data from primary objectives 1 and 2.

3. To investigate the relationship between anti-StrepA antibody titres and risk of incident colonisation and infection to explore serological correlates of protection.

Assessed by measurement of antibodies to streptococcal antigens combined with clinical data from primary objectives 1 and 2.
Assessed with mixed effects logistic regression and Cox Proportional Hazards Models

# Plants

Seed stocks

*Report on the source of all seed stocks or other plant material used. If applicable, state the seed stock centre and catalogue number. If plant specimens were collected from the field, describe the collection location, date and sampling procedures.*

Novel plant genotypes

*Describe the methods by which all novel plant genotypes were produced. This includes those generated by transgenic approaches, gene editing, chemical/radiation-based mutagenesis and hybridization. For transgenic lines, describe the transformation method, the number of independent lines analyzed and the generation upon which experiments were performed. For gene-edited lines, describe the editor used, the endogenous sequence targeted for editing, the targeting guide RNA sequence (if applicable) and how the editor was applied.*

Authentication

*Describe any authentication procedures for each seed stock used or novel genotype generated. Describe any experiments used to assess the effect of a mutation and, where applicable, how potential secondary effects (e.g. second site T-DNA insertions, mosiacism, off-target gene editing) were examined.*

## Flow Cytometry

### Plots

Confirm that:

☒ The axis labels state the marker and fluorochrome used (e.g. CD4-FITC).

☒ The axis scales are clearly visible. Include numbers along axes only for bottom left plot of group (a 'group' is an analysis of identical markers).

☒ All plots are contour plots with outliers or pseudocolor plots.

☒ A numerical value for number of cells or percentage (with statistics) is provided.

### Methodology

| | |
|---|---|
| Sample preparation | Myeloid leukaemia cell line THP-1 was purchased from ATCC (ATCC TIB-202) and expanded and maintained as per manufacturer's instruction. Prior to the beginning of the assay, undifferentiated cells in suspension were counted and checked for vitality >95% via NucleoCounter cell counter. Cells were then incubated with FITC-labelled bacteria or beads opsonized with human sera (as per methods) to induce opsonophagocytosis of bacteria or beads. |
| Instrument | BD Accuri C6 Plus flow cytometer with C-sampler Plus (Beckton Dickinson & Company). |
| Software | Samples were analyzed in FlowJo (Beckton Dickinson & Company, version 10.9.0). |
| Cell population abundance | Not applicable since no sorting was conducted. |
| Gating strategy | A gating is set in the forward (FSC) and side scatter (SSC) based to a THP-1 only control. In a typical sample, cells fall within the same gate and are still distinguishable as detached from the rest of events with monocytes typical FSC and SSC coordinates.<br><br>Detailed characterisation of the flowcytometric assay used in this manuscipt are provided in a referenced manuscript: https://doi.org/10.1016/j.jim.2024.113652 |

☒ Tick this box to confirm that a figure exemplifying the gating strategy is provided in the Supplementary Information.
